# Radiometals in Imaging and Therapy: Highlighting Two Decades of Research

**DOI:** 10.3390/ph16101460

**Published:** 2023-10-13

**Authors:** Shalini Sharma, Mukesh K. Pandey

**Affiliations:** Division of Nuclear Medicine, Department of Radiology, Mayo Clinic, Rochester, MN 55905, USA; sharma.shalini@mayo.edu

**Keywords:** positron emission tomography, single photon emission computerized tomography, imaging, radiometals, radionuclide therapy

## Abstract

The present article highlights the important progress made in the last two decades in the fields of molecular imaging and radionuclide therapy. Advancements in radiometal-based positron emission tomography, single photon emission computerized tomography, and radionuclide therapy are illustrated in terms of their production routes and ease of radiolabeling. Applications in clinical diagnostic and radionuclide therapy are considered, including human studies under clinical trials; their current stages of clinical translations and findings are summarized. Because the metalloid astatine is used for imaging and radionuclide therapy, it is included in this review. In regard to radionuclide therapy, both beta-minus (β^−^) and alpha (α)-emitting radionuclides are discussed by highlighting their production routes, targeted radiopharmaceuticals, and current clinical translation stage.

## 1. Introduction

External radiation therapy is a common and effective treatment for various cancers. Radiation therapy was first applied over 100 years ago, immediately after the discovery of the X-ray [1]. Clinicians used it for the treatment of skin diseases, lupus, and other lesions [2,3,4,5]. However, due to the collateral damage and resulting side effects, including hair loss, blurry vision, and dry and itchy skin, its application stalled and triggered the need for an alternative treatment option [6].

In the last two decades, nuclear medicine, including radionuclide therapy, has observed significant growth and development: newer radiopharmaceuticals have been introduced, and their applications in imaging and targeted radionuclide therapy have been diverse [7]. Targeted radionuclide therapy (TRT) has great potential to destroy even the smallest clusters of metastatic cancer cells present anywhere in the body, which is hard to achieve with either external beam radiation therapy or surgery. Therefore, TRT has been clinically used to treat numerous malignancies, such as neuroblastoma, along with breast, thyroid, and prostate cancer [7,8,9]. Treatments using TRT have been explored with and without additional treatment options, such as surgery and chemotherapy.

Typically, therapeutic radiopharmaceuticals consist of four components: (i) a therapeutic radioactive isotope (e.g., ^177^Lu, ^67^Cu, ^90^Y, ^212^Pb, ^212^Bi, ^225^Ac, ^223^Ra, etc.), (ii) a chelator, (iii) a linker, and (iv) a targeting vector, which delivers the isotope to the affected organs/tissues for treatment. The targeting vector could be a monoclonal antibody (mAb), antibody (Ab) fragment (diabody, nanobody, or single chain variable fragment), protein, aptamer, peptide, extracellular vesicle, virus, or simply a small molecule, such as an inhibitor [7]. Radiometal-based imaging and diagnostic radiopharmaceuticals can be categorized as (i) positron emission tomography (PET) radiopharmaceuticals (bioactive molecules labeled with positron-emitting [β^+^] isotopes) or (ii) single photon emission computed tomography (SPECT) radiopharmaceuticals (bioactive molecules labeled with gamma-emitting [γ] isotopes). Depending on the type of radiation-emitting isotopes, the therapeutic radiopharmaceuticals can be subdivided into (i) alpha (α) particle-emitting targeted radiopharmaceuticals, (ii) beta minus (β^−^) particle-emitting targeted radiopharmaceuticals, and (iii) Meitner−Auger electron (MAE)-emitting radiopharmaceuticals [10]. Additionally, when radiopharmaceuticals are radiolabeled with both the diagnostic (imaging) and therapeutic isotopes, which could use the same element (^64^Cu/^67^Cu, ^86^Y/^90^Y, ^44/43^Sc/^47^Sc) or different elements (^64^Cu/^212^Pb, ^68^Ga/^177^Lu, ^68^Ga/^223^Ra), they are called “theranostic radiopharmaceuticals.” Depending on the plasma half-life of the targeting vector and the application (imaging/therapy), an appropriate radionuclide should be selected for radiolabeling. This article covers advancements made in radiometal-based diagnostic and therapeutic radiopharmaceuticals in the last two decades.

## 2. Diagnostic Radionuclides

PET and SPECT are radionuclide-based imaging modalities used routinely in nuclear medicine practice that fall under the category of “molecular imaging” because their radiotracers provide information about particular biological processes at the cellular and molecular levels.

(i) PET measures the energy produced by the two gamma photons (511 keV) that result from annihilation of the positron emitted from the PET radionuclide with atomic electron [11]. The emitted gamma photons are detected with γ-cameras, also called scintillation detectors, which produce reconstructed three-dimensional images depicting the spatial distribution of radiotracers [11]. The common examples of PET probes includes [^18^F]FDG, [^13^N]NH_3_, [^68^Ga]Ga-PSMA, and [^18^F]NaF. Preclinical animal PET and clinical PET scanners offer spatial resolution of 1–2 mm and 6–10 mm, respectively, with high sensitivity of 10^−11^–10^−12^ mol/L. This level of sensitivity is sufficient to detect biological changes in an organ or tissue to identify the onset of a disease before anatomical changes occur [12].
(ii) SPECT measures the single gamma photons emitted directly from γ-emitting radionuclides called SPECT radiopharmaceuticals. The conventional clinical SPECT scanners have lower sensitivity (10^−10^–10^−11^ mol/L) and lower spatial resolution (7–15 mm) compared to PET scanners due to the limited performance of collimators [12,13,14]^.^ Despite this, SPECT is the most routinely used nuclear imaging procedure in the clinic and is less expensive compared to PET. The most common SPECT isotopes are ^111^In, ^99m^Tc, ^123/131/125^I, and ^67^Ga.

Recent advances in SPECT γ-cameras, collimators, and reconstruction algorithms have enhanced the spatial resolution and sensitivity of SPECT scanners, allowing for the imaging of a wide range of isotope energy (20–300 keV) [15,16]. Nevertheless, both PET and SPECT need either computed tomography (CT) or magnetic resonance imaging (MRI) for accurate anatomical information. Interestingly, PET cannot distinguish between two different PET probes when injected simultaneously because it measures two γ-rays with the same energy (511 keV); meanwhile, SPECT does have multiplexing capabilities because each radionuclide produces different γ-rays, enabling it to image different targets simultaneously [17].

## 3. Therapeutic Radionuclides

As stated previously, therapeutic radionuclides emit α particles, β^−^ particles, and/or low-energy MAEs (non-energetic particles).

**Figure 1:**
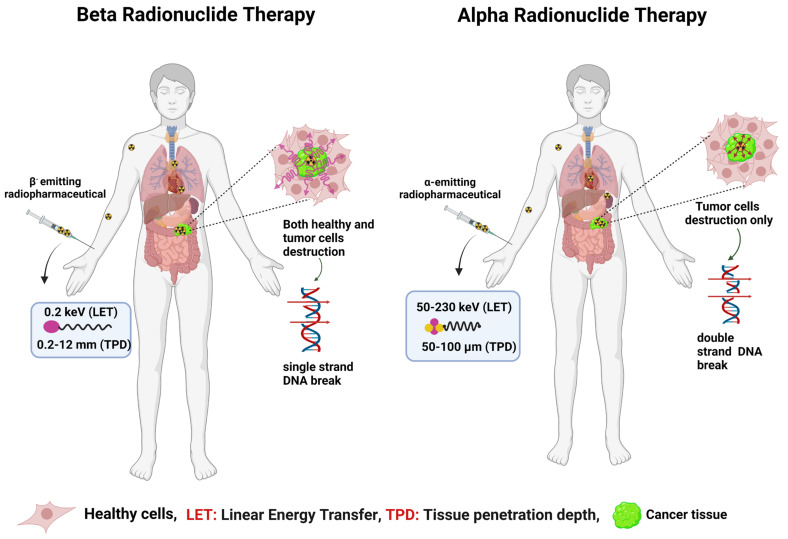
Comparison of beta and alpha radionuclide therapies.

(i) Beta minus emitters can be either of a high energy (^90^Y, E_β_^−^_max_ = 2.28 MeV,) or a low energy (^177^Lu, E_β_^−^_max_ = 496 keV), with tissue penetration ranges between 12 mm and 1.5 mm, respectively [18]. Given the long penetration depth of 0.2–12 mm and the moderate linear energy transfer (LET) radiation of ~0.2 keV/µm, β^−^ emitters are more suited to treating large-sized tumors (>0.5 cm), and they are considered the current gold standard in targeted radionuclide therapy [19,20].
(ii) Alpha emitters emit α particles with high LET energies of 50–230 keV/µm and shorter penetration depths of 50–100 µm (i.e., 5–10 cell diameters) [21]. Alpha radionuclide-based targeted therapy is called targeted alpha therapy (TAT), and it is well suited for the treatment of hematological disease, small tumors, metastasis, and isolated cancer cells. Alpha emitters are perceived as a better therapeutic alternative to beta emitters due to their high LET and short tissue penetration range.
(iii) Meitner−Auger electrons are low-energy electrons that can penetrate up to the subcellular nanometer range (<0.5 µm), resulting in a high LET of 4–26 keV/µm [22]. Given the low tissue penetration range and high LET in an extremely small area, MAE emitters could be highly valuable for treating metastatic cancers if delivered selectively within the nucleus of the cancer cells [22]. Figure 1 explains the difference between LET, pathlength (penetration range), and the usefulness of α and β^−^ radionuclide therapies.

## 4. Positron Emitters

### 4.1. Radioisotopes of Copper

#### 4.1.1. General Information

Among the long list of radioisotopes of copper (Cu), ^60^Cu, ^61^Cu, ^62^Cu, and ^64^Cu are used for diagnostic imaging, while ^64^Cu and ^67^Cu are applied in radionuclide therapy [23]. ^64^Cu decays by both β^+^ (~17%) and β^−^ (~38%), making it applicable for PET imaging and targeted radionuclide therapy; therefore, it is considered a theranostic radionuclide [24]. In addition, ^64^Cu also decays by electron capture (EC), which results in a cascade of Auger electrons [22,24]. The decay characteristics of Cu radioisotopes are mentioned in Table 1.

#### 4.1.2. Growth and Advancement of Radiopharmaceuticals Labeled with Copper-Radioisotopes in Clinical Practice

In 1997, [^62^Cu]Cu-diacetylbis(4-methylthiosemicarbazone), also known as [^62^Cu]Cu-ATSM, was first discovered as a hypoxia imaging agent in a rat model of cardiac ischemia [26]. Later, other Cu isotopes, including ^60/61/64^Cu, were used to radiolabel ATSM and employed for imaging of hypoxic solid tumors, with a similar uptake and clearance profile in patients [27,28,29,30,31].

Considering the longer half-life of ^64^Cu, [^64^Cu]Cu-ATSM was applied as a hypoxia imaging radiotracer in rectal cancer (National Clinical Trial (NCT) 03951337) [32]. However, several debatable preclinical studies highlighted its lower uptake in hypoxic tumors [28]. The therapeutic potential of [^64^Cu]Cu-ATSM was first studied in 2001, where it resulted in a six-fold increase in the survival of 50% of hamsters bearing human GW39 colon cancer [33]. Later, several additional preclinical studies supported the theranostic potential of [^64^Cu]Cu-ATSM to treat various colon carcinoma xenografts (Colon-26, HT-29). In addition to preclinical studies, clinical studies are needed to prove its true theranostic value [34,35,36].

During the last two decades, various ^64^Cu-labeled Abs have been developed for immuno-PET imaging [37,38,39]. Among them, [^64^Cu]Cu-DOTA-trastuzumab showed promising clinical utility in identifying HER2+ tumors with high sensitivity (~89%) in breast cancer patients [40,41].

Currently, commonly used somatostatin radiotracers for neuroendocrine tumor (NET) diagnosis are [^111^In]In-DTPA-octreotide [42], [^99m^Tc]Tc-EDDA/HYNIC-TOC [43], and [^68^Ga]Ga-DOTATOC [44], with first-in-human studies reported in the years 1993, 2005, and 2001, respectively. During 2012–2017, Pfeifer and Johnbeck’s team conducted two separate clinical studies using newly developed [^64^Cu]Cu-DOTA-TATE on NET patients. Their findings revealed the outperformance of [^64^Cu]Cu-DOTA-TATE over [^111^In]In-DTPA-octreotide [45] and [^68^Ga]Ga-DOTATOC [46] in terms of spatial resolution, lesion detection rate, and, most importantly, the ability to identify additional lesions.

A recent clinical study of [^64^Cu]Cu-DOTA-TATE (200 MBq dose) demonstrated that [^64^Cu]Cu-DOTA-TATE is excellent for lesion detection in neuroendocrine neoplasm patients [47]. In 2020, the Food and Drug Administration (FDA) approved the first ^64^Cu-labeled PET radiopharmaceuticals, [^64^Cu]-DOTA-TATE (Detectnet^TM^), for the localization of somatostatin-targeting receptor (SSTR)-positive NETs in adult patients [48]. Additionally, the radiotracer [^67/64^Cu]Cu-Sar-TATE was recently entered into multiple clinical trials to diagnose and treat SSTR-positive tumors [49].

In addition to radiolabeled somatostatin-targeting peptides, a series of PSMA ligands have been identified and radiolabeled with Cu radioisotopes for clinical diagnosis and radionuclide therapy applications in PCa [50,51,52]. In 2016, ^64^Cu-labeled PSMA-617 became the first ^64^Cu-labeled ligand for PET imaging of PCa patients and was investigated at two nuclear medicine centers (Vienna, Austria, and Bed Berka, Germany) [53]. Even though [^68^Ga]Ga-PSMA is an excellent tracer to detect PCa and metastatic lesions in the lymph node or bone at low PSA levels [54], the advantage of the lower positron energy of ^64^Cu (E_β_^+^_avg_ = 278 keV) vs. ^68^Ga (E_β_^+^_avg_ = 829 keV) and the longer half-life (12.7 h) of ^64^Cu allow its distribution and use as [^64^Cu]Cu-PSMA-617 at various clinical PET centers with no sophisticated onsite radiotracer production facility [53].

^67^Cu is one of the most promising radionuclides for radioimmunotherapy (RIT), as its 61.8 h isotopic half-life is well matched with the residence time of a typical Ab on the tumor site. In 1998, DeNardo reported a pilot study of [^67^Cu]Cu-2IT-BAT-Lym-1 to image and treat chemo-resistive B-cell in non-Hodgkin’s lymphoma while employing favorable SPECT imaging and the remarkable radiotherapeutic effects of ^67^Cu-labeled 2IT-BAT-Lym-1 [55]. The clinical investigation of Cu radiopharmaceuticals is outlined in Table 2.

#### 4.1.3. Production and Availability

At present, the most common method to produce ^64^Cu is proton irradiation of enriched ^64^Ni via a ^64^Ni(p,n)^64^Cu nuclear reaction in a small−medium-energy biomedical cyclotron [66]. The main route to produce ^67^Cu for decades had been via a ^68^Zn(p,2p)^67^Cu nuclear reaction that utilizes enriched ^68^Zn and high-energy proton irradiation (up to 40 MeV), which also coproduces ^64^Cu [67]. Recently, Mou et al. developed and patented the fabrication of multi-layer targets composed of enriched ^70^Zn and ^68^Zn that could maximize ^67^Cu production yield [68].

### 4.2. Radioisotopes of Gallium

#### 4.2.1. General Information

Among the many radioisotopes of Gallium (Ga), ^66^Ga, ^67^Ga, and ^68^Ga are predominantly used in medical applications for the radiolabeling of various biomolecules [69]. ^67^Ga and ^68^Ga are predominantly used in nuclear medicine for SPECT and PET imaging, respectively. ^66^Ga ((t_1/2_ = 9.49 h) is an attractive PET radionuclide with a relatively longer half-life than ^68^Ga (t_1/2_ = 67.71 min). Due to its high positron emission energy (E_β+avg_ = 1750 keV), though, along with the co-emission of higher gamma rays than ^68^Ga, ^66^Ga suffers from poor image resolution and high radiation exposure to workers, limiting its medical application [70].

^67^Ga is one of the longer-lived Ga radioisotopes, and it decays by EC (100%) with multiple gamma emissions, with the most common gamma energies emitted as 93 keV (39%), 184 keV (21%), and 300 keV (17%) for the SPECT imaging [71]. [^67^Ga]Ga-citrate is the most popular radiopharmaceutical of ^67^Ga. For several decades, it has been used in the diagnosis of osteomyelitis and other bone infections [72,73]. To date, [^67^Ga]Ga-citrate scintigraphy is used worldwide for the diagnosis of lymphomas [74], lung cancer [75,76], and inflammation of the kidneys [77]. The nuclear decay properties of Ga radionuclides are displayed in Table 3.

#### 4.2.2. Clinical Practice

Gallium-68 is one of the earliest radionuclides applied in the early days of PET scans (early 1960s), long before the discovery of [^18^F]fluorodeoxyglucose (FDG) in 1978 [79]. However, the growth of ^68^Ga-labeled radiopharmaceuticals in clinical applications began after the commercial launch of next-generation ^68^Ge/^68^Ga generators in the early 21st century (mid-2000s) [80,81]. During 2000–2010, various Ga-68-labeled peptide-based radiopharmaceuticals, such as [^68^Ga]Ga-DOTA-TATE [82], [^68^Ga]Ga-DOTA-TOC [44,82], and [^68^Ga]Ga-DOTA-NOC [83], were clinically evaluated in peptide receptor radionuclide therapy (PRRT) to visualize NET-expressing SSTR2. Later, [^68^Ga]Ga-PSMA-11 (also known as PSMA-HBED, HBED-CC) was investigated for the diagnosis of recurrent PCa, and it received FDA approval in 2020 [84]. Several clinical studies involving ^68^Ga were recently conducted with its “theranostic twin”, ^177^Lu, for diagnosis and radionuclide therapy of NETs and PCa. A tremendous growth in the application of [^68^Ga]Ga-PSMA-11 has occurred for imaging metastatic castration-resistant PCa over other radiotracers. Additionally, there is high demand for its theranostic pair of PSMA, labeled with either beta emitter ^177^Lu or alpha emitter ^225^Ac [85,86,87,88]. The clinical investigation of ^68^Ga radiopharmaceuticals is noted in Table 4.

#### 4.2.3. Production and Availability

Currently, the most convenient method to produce ^68^Ga is from germanium (Ge) 68 (t_1/2_~271 d) using ^68^Ge/^68^Ga generators [89]. However, the shortage of these generators and on-demand supply of ^68^Ga have led to the generation of alternative methods of production using, as an example, cyclotrons (12–17 MeV) via the ^68^Zn(p,n)^68^Ga nuclear reaction [90]. During 2014–2019, Pandey et al. pioneered cyclotron-mediated ^68^Ga production using a liquid target to overcome the global shortage of ^68^Ga [91,92,93]. Besides liquid target-based production, several high-yielding solid target-based production methods, also using cyclotron, have been developed and commercialized to meet the upcoming demands of ^68^Ga [94,95,96]. On the other hand, the common production of ^67^Ga through the irradiation of ^nat^Zn or isotopically enriched ^68^Zn targets via ^68^Zn (p,2n)^67^Ga or ^67^Zn(p,n)^67^Ga on cyclotron have been reported [97].

**Table 4:** Clinical applications of ^68^Ga-labeled radiopharmaceuticals.

Radiopharmaceuticals	Biological Target	NCT Number ^	Disease
[^68^Ga]Ga-PSMA-11	PSMA	NCT03207139 (Phase II; completed) NCT03982407 (Early Phase I; completed)	Latent prostate cancer [98], hepatocellular carcinoma [99]
[^68^Ga]Ga-NGUL/[^177^Lu]Lu-DGUL	PSMA	NCT05547061 (Phase I/II; ongoing)	Metastatic castration-resistant prostate cancer [100]
[^68^Ga]Ga-DOTA-TATE vs. [^68^Ga]Ga-DOTA-TOC	SSTR2	NCT04298541 (Phase II; ongoing)	Meningioma [101]
[^68^Ga]Ga-DOTA-TOC	SSTR	NCT02441062 (Phase II; completed)	Neuroendocrine tumors [102]
[^68^Ga]Ga-FAPi-46	FAPI	NCT04457258 (Early Phase I; ongoing)	Sarcoma, recurrent or metastatic, sarcoma [103]

NCT: National clinical trial, NETs: Neuroendocrine tumors, PSMA: Prostate-specific membrane antigen, SSTR2: Somatostatin receptor 2, FAPI: Fibroblast activation protein inhibitor, ^ clinicaltrials.gov and data accessed on 15 August 2023.

### 4.3. Radioisotopes of Zirconium

#### 4.3.1. General Information

Zirconium-89 (^89^Zr) is a promising radionuclide for the PET imaging of Abs due to its longer physical half-life (78.4 h), which matches with the blood half-life of most full-length Abs (days to weeks) [104]. ^89^Zr has a relatively short penetration range by emitting low-energy positrons (E_β_^+^_avg_ = 396 keV), which facilitate high-resolution PET images [104]. However, ^89^Zr emits an abundance of high-energy γ-rays of 909 keV, adding radiation exposure to medical staff and patients [104]. Table 5 summarizes the decay characteristics of Zr-89.

**Table 5:** Decay characteristics of Zirconium-89 ^#^.

Isotope	Half-Life (t_1/2_)	Decay Characteristics	Energy E_β_^+^_avg_ (keV)	E_γ_; keV (Intensity%)
^89^Zr	78.41 h	β^+^ = 22.3%	395.5	511 (45.5)
		EC = 76.6%		909.2 (99)

^#^ Data on ^89^Zr are from [105]. Please refer to decay Scheme 1A.

#### 4.3.2. Clinical Practice

In 2006, the first clinical study of [^89^Zr]Zr-immuno PET was reported, where ^89^Zr-labeled chimeric mAb U36 localized in all primary tumors and lymph node metastasis of head and neck cancer patients with an accuracy as high as 93% [106]. Presently, the FDA has approved hundreds of mAbs against various biological targets, such as HER2, CD20, epidermal growth factor receptor (EGFR), vascular endothelial growth factor (VEGF), and PSMA, resulting in several [^89^Zr]Zr-Immuno PET-based clinical oncology trials [107,108]. Currently, [^89^Zr]Zr-trastuzumab and [^89^Zr]Zr-pertuzumab are the two common choices for immuno-PET-targeting HER2+ breast cancer. The first-in-human studies using [^89^Zr]Zr-trastuzumab (37 MBq) and [^89^Zr]Zr-pertuzumab (74 MBq) on metastatic breast cancer patients were reported in the years 2010 and 2018, respectively [109,110]. Currently, both radiopharmaceuticals are registered in clinical trials.

Additional clinical pilot studies have been published using [^89^Zr]Zr-immuno-PET probes, such as [^89^Zr]Zr-bevacizumab targeting VEGF-A expression [111], [^89^Zr]Zr-rituximab targeting B-lymphocyte antigen (CD20) expression [112], and [^89^Zr]Zr-cetuximab-targeting EGFR [113], in various tumors. These [^89^Zr]Zr-immuno-PET probes have been shown to be useful for imaging and/or radionuclide therapy applications. Currently, [^89^Zr]Zr-bevacizumab is registered in an ongoing clinical trial (National Clinical Trial (NCT) 01894451) [114]. Considering the long blood circulation time of monoclonal Abs (mAbs), alternative Abs and their fragments were developed in the last five years to significantly shorten their retention time in blood and to rapidly clear the unbound fragments from the body [115]. Early examples of Ab fragment application are minibody-based radiopharmaceuticals, such as [^89^Zr]Zr-Df-IAB2M, which were used to detect PSMA-positive PCa and recurrent cerebral high-grade gliomas [116,117]. In addition to minibody-based radiopharmaceuticals, several preclinical studies reported ^89^Zr-labeled affibodies, such as [^89^Zr]Zr-Df-ZEGFR:03115 and [^89^Zr]Zr-DFO-MAL-Cys-MZ, as targeting EGFR and HER2, respectively [118,119]. To fully evaluate the clinical value of affibodies and Ab fragments, additional clinical studies demonstrating their usefulness are paramount.

Besides Abs, cell labeling with ^89^Zr has been explored for the imaging of white blood cells and CAR-T cells. Several preclinical studies using [^89^Zr]Zr-oxine and [^89^Zr]Zr-Df-aTCRmu-F(ab’)_2_ were reported to track T cells in glioblastoma and acute myeloid sarcoma, respectively [120,121]. Covalent tethering of [^89^Zr]Zr-DBN to cells is another highly studied methodology to noninvasively track various cell types with PET. Published reports of this method demonstrate that it offers a robust and reliable approach that could be translated in humans for monitoring cell-based therapies [122,123,124,125]. Table 6 summarizes the clinical applications of ^89^Zr-based radiopharmaceuticals.

#### 4.3.3. Production and Availability

The production and availability of ^89^Zr have been improved significantly in the last two decades. Various methods of ^89^Zr production on solid and liquid targets using cyclotron have evolved over the years, resulting in better and simplified methods of purification and radiolabeling [91,93,126,127,128,129,130,131,132]. The main route of production is proton irradiation of yttrium (Y) via a ^89^Y(p, n) ^89^Zr nuclear reaction [126]. At present, ^89^Zr is routinely produced at various academic institutions for their own use, including Mayo Clinic Rochester, and for supplying other institutions, including the University of Wisconsin, the University of Alabama, and commercial vendors within the United States. Some European and Asian academic institutions also manufacture ^89^Zr routinely and use it predominantly in preclinical studies. In the last 10 years, several groups have come up with alternative solutions that facilitate the GMP-grade production and formulation of ^89^Zr. For example, Wooten et al. designed an automated system for routine ^89^Zr production and purification at high radioactivity quantities, with >99.9% of radionuclidic purity [133]. In terms of purification, Pandey et al. developed a simplified synthesis of hydroxamate resin for trapping of Zr-89 with a trapping efficiency of 93% and its subsequent elution either as oxalate or phosphate in a high elution efficiency (>90%) [134]. Recently, the same group designed a new solid target insert and optimized the thickness of ^89^Y foil and proton beam energy to improve the production yield of ^89^Zr (~129 mCi or 4.77GBq) using medium energy cyclotrons [126]. Others in the field have also significantly contributed towards the advancement of Zr-89 production and purification [135,136].

**Table 6:** Clinical applications of ^89^Zr-labeled radiopharmaceuticals currently under investigation.

Radiopharmaceuticals	Targets	NCT Number ^	Disease
[^89^Zr]Zr-Df-hJ591	PSMA	NCT01543659 (Phase I/II; ongoing)	Prostate cancer [137]
[^89^Zr]Zr-Df-IAB2M	PSMA	NCT02349022 (Phase II; completed)	Prostate cancer [138]
[^89^Zr]Zr-Df-IAB22M2C	CD8^+^ Tlymphocytes	NCT05013099 (Phase IIb; ongoing) NCT03107663 Phase I; completed) NCT03802123 (Phase II; completed)	Melanoma [139] Renal cell carcinoma [140] Metastatic solid tumors [141]
[^89^Zr]Zr-daratumumab	CD38	NCT03665155 (Phase II; completed)	Multiple myeloma [142]
[^89^Zr]Zr-trastuzumab	HER2+	NCT01420146 (Phase I; completed)	Breast neoplasm [143]
[^89^Zr]Zr-ss-pertuzumab	HER2-	NCT04692831 (Phase I; ongoing)	Breast carcinoma [144]
[^89^Zr]Zr-bevacizumab	VEGF	NCT01894451 (Early Phase I; completed)	Inflammatory breast carcinoma [114]
[^89^Zr]Zr-panitumumab	EGFR	NCT03733210 (Phase I; completed)	Carcinoma of head and neck [145]
[^89^Zr]Zr-cetuximab	EGFR	NCT00691548 (Phase I; completed)	Stage IV cancer [146]
[^89^Zr]Zr-girentuximab	Carbonic anhydrase	NCT03849118 (Phase III; completed)	Renal cell carcinoma [147]
[^89^Zr]Zr-durvalumab	PDL-1	NCT03853187(Phase II; completed)	Non-small cell lung cancer [148]
[^89^Zr]-DFO-atezolizumab	PDL-1	NCT04006522 (Phase II; ongoing)	Renal cell carcinoma [149]

NCT: National clinical trial, PSMA: Prostate-specific membrane antigen, CD38: Cluster of differentiation 38, HER2: Human epidermal growth factor 2, VEGF: Vascular endothelial growth factor, EGFR: Epidermal growth factor receptor, PDL-1: Programmed cell death ligand-1, ^ clinicaltrials.gov and data accessed on 15 August 2023.

### 4.4. Radioisotopes of Scandium

#### 4.4.1. General Information

Scandium (Sc) has 25 different radioisotopes, but ^43^Sc, ^44^Sc, and ^47^Sc are the commonly explored radionuclides for PET imaging and targeted radionuclide therapy applications [150]. ^44^Sc and ^43^Sc are promising PET radionuclides, and they are superior alternatives to ^68^Ga because of their lower positron energy and almost 3.5-fold longer half-life [150,151]. However, ^44^Sc also decays via high gamma ray (E_γ_ = 1157 keV; 99.9% abundance) emission and could give a high radiation exposure dose compared to other competing PET radionuclides [152]. The decay properties of Sc radionuclides are given in Table 7.

#### 4.4.2. Current Clinical Application of Scandium-44

In 2017, the first clinical study of generator-derived ^44^Sc with [^44^Sc]Sc-DOTATOC was reported for the imaging of a metastatic neuroendocrine neoplasm at Bed Berka [153]. Recently, [^44^Sc]-PSMA-617 was also applied for the imaging of PCa patients [154], and it showed performance comparable to [^68^Ga]Ga-PSMA-617 in terms of tumor uptake and image quality [154]. Given the availability of both imaging (^43/44^Sc) and therapeutic (^47^Sc) radionuclides, Sc radiopharmaceuticals are gaining significant interest as an alternative theranostic pair [155].

^43^Sc is another PET isotope of the Sc family with similar physical characteristics to ^44^Sc, but it is devoid of high-energy gamma emission and lower positron energy (E_β_^+^_avg_ = 476 keV), making it a more favorable imaging isotope than ^44^Sc [150]. However, no preclinical or clinical studies are yet reported with ^43^Sc-labeled radiopharmaceuticals.

^47^Sc is a radio theragnostic isotope that emits low-energy β^−^ particles (E_β_^−^_avg_ = 162 keV) and low-energy γ-radiations (E_γ_ = 159 keV) [150]. The decay characteristics of ^47^Sc are like ^67^Cu (E_β-avg_ = 141 keV, E_γ_ = 184 keV) and ^177^Lu (E_β_^−^_avg_ = 134 keV, E_γ_ = 113, 208 keV). Recently, a comparative preclinical study with [^47^Sc]Sc-folate (12.5 MBq), [^177^Lu]Lu-folate (10 MBq), and [^90^Y]Y-folate (5 MBq) showed a similar therapeutic response in an ovarian xenograft model [156]. Due to the challenging production routes of ^47^Sc, though, clinical studies involving ^47^Sc have yet to evolve [156].

#### 4.4.3. Production and Availability

In 2010, Frank Rosch et al. reported the production of ^44^Sc (approx.185 MBq) using a ^44^Ti/^44^Sc generator for the first time at Bed Berka, Germany [157]. However, the production of the parent radionuclide, ^44^Ti, and the accessibility of these generators were challenging. Later, in 2015, Van der Meulen et al. reported a cyclotron-based production of ^44^Sc via proton irradiation (11 MeV) of an enriched ^44^Ca target, which allowed elution of approximately 2 GBq of ^44^Sc at the Paul Scherrer Institute (PSI) in Switzerland [158]. Later, Szkliniarz et al. reported several cyclotron-based production routes for emerging ^43^Sc using either α particles or deuteron beams because the limited availability of high-energy multi-particle cyclotrons restricted the utility of these production routes [159]. Van der Meulen et al. demonstrated the production of 480 MBq of ^43^Sc using enriched ^43^CaCO_3_ and targets via a ^43^Ca(p,n)^43^Sc reaction; limited purity of ^43^Sc was obtained due to the co-produced mixture of ^43^Sc (66.2%) and ^44^Sc (33.3%) [160]. ^47^Sc can be produced using a cyclotron [161], neutron flux reactor [162], or electron linear accelerator [163,164].

### 4.5. Radioisotopes of Terbium

#### 4.5.1. General Information

Among the various radioisotopes of Terbium (Tb), four (^149/152/155/161^Tb) are of great interest in nuclear medicine and are commonly referred as a “Swiss army knife” of nuclear medicine [165]. ^149^Tb has both positron and α-emission properties for both PET and targeted therapy applications [166]. ^152^Tb is another positron emitter with a relatively longer half-life of 17.5 h that could be utilized for radiolabeling of large biomolecules [166]. The decay characteristics of Tb radionuclides are listed in Table 8.

In 2012, Muller et al. performed the first radiolabeling of albumin-binding folate conjugates (cm09) with ^149/152/155/161^Tb in an FR-positive tumor xenograft mouse model [167]. The findings demonstrated excellent tumor visualization through PET/CT using [^152^Tb]Tb-cm09 and SPECT/CT, using both [^155^Tb]Tb-cm09 and [^161^Tb]Tb-cm09 probes at 24 h post administration. On the other hand, α therapy version [^149^Tb]Tb-cm09 and β^−^ therapy version [^161^Tb]Tb-cm09 resulted in significantly delayed tumor growth by 33% and 80%, respectively.

#### 4.5.2. Preclinical and Clinical Applications

(i) ^149^Terbium: ^149^Tb represents one of the powerful candidates for TAT, which emits short penetrating (~25 µm range) α particles (Eα = 3.97 MeV; I_α_ = 16.7%) compared to currently employed α emitters [168]. Because ^149^Tb also decays by positrons (β^+^) and γ-radiations, ^149^Tb-labeled radiopharmaceuticals could also be useful for PET and SPECT imaging [168].

In 2004, Beyer et al. demonstrated the first preclinical RIT with [^149^Tb]Tb-rituximab in a leukemia xenograft mouse model, which resulted in tumor-free survival >120 days among 89% of the [^149^Tb]Tb-rituximab-treated mice [169]. However, nearly 28% of the residual radioactivity of longer-lived daughter nuclides, ^149^Eu (t_1/2_ = 93 d),^145^Sm (t_1/2_ = 340 d), and others were retained mainly in the mice bone marrow. Baum et al. demonstrated the first in-man PET/CT study of SSTR-targeted [^149^Tb]Tb-DOTANOC on a male patient diagnosed with neuroendocrine ileum. The result was an excellent localization of [^149^Tb]Tb-DOTANOC in this neuroendocrine neoplasm, in addition to multiple lymph nodes, skeletal metastasis, and SSTR-expressing organs [170].

Currently, ^149^Tb is predominantly produced by ISOLDE/CERN (Switzerland), TRIUMF (Vancouver, Canada), and PNPI (Gatchina, Russia) [171]. More recently, preclinical studies with [^149^Tb]Tb-DOTANOC and [^149^Tb]Tb-PSMA-617 were reported in tumor xenograft mouse models of AR42J pancreatic and prostate cancers, respectively [168,172]. Thus far, no clinical study has been reported with ^149^Tb [168].

(ii) ^152^Terbium: ^152^Tb is a diagnostic radionuclide that decays via positron emission (E_β_^+^_avg_ = 1142 keV) and multiple gamma radiations, which could lead to high radiation exposure [170]. The relatively long half-life of ^152^Tb (t_1/2_ = 17.5 h) allows it to be useful in dosimetry estimation. In fact, ^152^Tb is an exact diagnostic match for ^149^Tb and ^161^Tb, as well as other clinically useful therapeutic radionuclides, like ^177^Lu, due to their similarities in coordination chemistry and pharmacokinetics.
(iii) ^155^Terbium: ^155^Tb is a suitable SPECT isotope, a promising alternative to the ^111^In isotope, and it could be useful for dosimetry estimation of β^−^ emitters, like ^177^Lu, ^90^Y, and ^166^Ho [173].
(iv) ^161^Terbium: ^161^Tb decays by low-energy (E_β_^−^_avg_ = 154 keV) (β^−^) emission, having a short tissue penetration (0.29 mm) range and a long half-life (t_1/2_) of 6.8 d [174]. The decay characteristics and half-life of ^161^Tb are like ^177^Lu (E_β_^−^_avg_ = 134 keV, t_1/2_ = 6.7d) [174], although ^161^Tb also emits a substantial number of auger electrons, which could be advantageous for therapeutic applications. However, the clinical superiority of ^161^Tb over ^77^Lu is yet to be established [175,176,177]. In addition to radionuclide therapy, ^161^Tb also emits gamma photons enabling SPECT imaging [174]. Recently, Baum et al. demonstrated the first-in-human SPECT imaging using [^161^Tb]Tb-DOTATOC in patients with paraganglioma and NETs and showed high-quality images and visualization of hepatic metastasis as well as multiple osteoblastic skeletal metastasis in patients [178].

The main constraint to the wider application of Tb isotopes is their availability: there are insufficient production quantities. The production of Tb isotopes requires expensive enriched targets and accelerator-based isotope separation on-line technology (ISOLDE), which is not widely available [166]. Table 9 summarizes the clinical investigation of Tb radionuclides.

#### 4.5.3. Production and Availability

In 2012, Muller et al. reported on the production of ^149/152/155^Tb in a range of ~6–15 MBq activity through a high-energy proton-induced spallation of tantalum foil targets, followed by dissolution and isotope separation [167]. Such a high-energy proton accelerator facility and mass separation technology (ISOLDE) are limited to a few centers worldwide, including CERN, Switzerland. Lately, CERN-MEDICIS (Medical isotopes collected from ISOLDE) technology was developed, which allowed for the production of 38 GBq of ^149^Tb, 37 GBq of ^152^Tb, and 5.3 GBq of ^155^Tb [166]. ^161^Tb (up to 15 GBq) can be produced in a neutron flux reactor using ^160^Gd targets, as proposed by Lehenberger et al. at PSI, Switzerland [181]. Interestingly, the production concept and the cost of ^161^Tb is like a non-carrier added ^177^Lu [181].

### 4.6. Radioisotopes of Zinc

#### 4.6.1. General Information

Zinc (Zn) exists in three positron-emitting isotopes (^62/63/65^Zn) that have the potential to be used as PET biomarkers of zinc trafficking in various pathological conditions [182]. Among them, ^62^Zn has limited use because it decays to another positron-emitting isotope, ^62^Cu (β^+^ = 98%; t_1/2_ = 9.7 min), which could confound the image interpretation of PET scans [182]. Nevertheless, ^62^Zn has been used preclinically to image zinc transport in pancreatic exocrine function [183].

Among the Zn PET isotopes, ^65^Zn has the longest half-life (t_1/2_ = 243.9 d), making it unsuitable for diagnostic imaging because it will cause high radiation exposure to patients over time [182]. ^63^Zn has a favorable decay characteristic (β^+^ = 93%; t_1/2_ = 38.47 min) for diagnostic imaging and pharmacokinetic studies [184,185]. The decay properties of Zn radionuclides are given in Table 10.

#### 4.6.2. Clinical Applications

In 2016, DeGrado et al. conducted the first-in-human PET imaging study of [^63^Zn]Zn-citrate on Alzheimer’s disease patients [185]. Although low uptake of [^63^Zn]Zn-citrate was seen in the brain (SUV ~0.4) compared to other organs, like the liver, pancreas, kidney, and gastrointestinal tract, it was sufficient to study ^63^Zn clearance kinetics on a regional basis in those patients. The regions with slower ^63^Zn clearance corresponded to the regions of known amyloid-β pathology on [^11^C]C-PiB PET scans and also the regions of lower uptake on [^18^F]FDG-PET scans [185]. Further imaging studies are warranted, though, to study zinc homeostasis in persons with Alzheimer’s disease.

#### 4.6.3. Production and Availability

In 2014, DeGrado et al. developed a cyclotron-based production of ^63^Zn via a ^63^Cu(p,n)^63^Zn nuclear reaction using a liquid target by irradiating an isotopically enriched solution of [^63^Cu]Cu-nitrate [184]. ^63^Zn was produced with a specific activity of 41.2 + 18.1 MBq/µg (uncorrected) and radionuclidic purity of 99.9% using 1.23 M of [^63^Cu]-copper nitrate.

## 5. SPECT Probes

### 5.1. Technetium-99m

#### 5.1.1. General Information

Technetium-99m (^99m^Tc) is the most widely used medical isotope in nuclear medicine, accounting for more than 80% of all nuclear medicine procedures, including myocardial perfusion imaging, cancer, and infection imaging [186]. ^99m^Tc-based agents are a favored choice for cardiac imaging in the U.S [187]. ^99m^Tc mainly disintegrates into its other isomeric ^99^Tc (which is radioactive) with the release of low-energy monochromatic gamma rays (140.5 keV, 98.6%) that can be detected by any sensitive gamma cameras [188]. Despite the advent of superior PET technology and the prevalence of CT or MRI over nuclear medicine, ^99m^Tc-based radiopharmaceuticals have been continuously supplied in hospitals during routine clinical examinations [188]. The advantages behind them are (i) a short/sufficient half-life of 6 h, which offers minimum radiation exposure to patients, (ii) instant kit-based labeling and formulations due to rich coordination chemistry of Tc (multiple oxidation states), (iii) availability of transportable generators (^99^Mo/^99m^Tc) for production, and (iv) cost-effective SPECT gamma cameras compared to expensive PET technology. These points have solidified the continuous application of ^99m^Tc-labeled radiopharmaceuticals [188,189]. The nuclear decay characteristics of ^99m^Tc are given in Table 11.

#### 5.1.2. Clinical Applications

Among the various clinical applications of ^99m^Tc-labeled radiopharmaceuticals, [^99m^Tc]Tc-HYNIC-TOC (Tektrotyd) is commercially available for the imaging of metastatic NETs [190]. A recent comparative study of [^68^Ga]Ga-DOATATE and [^99m^Tc]Tc-HYNIC-TOC (^99m^Tc-octreotide) on NET patients showed the superiority of [^68^Ga]Ga-DOATATE over [^99m^Tc]Tc-HYNIC-TOC in terms of sensitivity and specificity [191]. However, it is reasonable to re-evaluate the performance of ^99m^Tc-radiopharmaceuticals using ultra-fast SPECT scanners that may increase the image resolution up to twofold [192]. Additionally, [^99m^Tc]Tc-MIP1404 and [^99m^Tc]Tc-MIP-1405 are the first [^99m^Tc]Tc-labeled PSMA ligands applied in humans [193]. Although both agents can visualize PSMA tumors and metastatic lymph node/bone lesions, [^99m^Tc]Tc-MIP1404 (also known as Tc-Trofolastat) is advantageous over [^99m^Tc]Tc-MIP-1405 to detect PCa at early stages of the disease, and it is currently registered in a phase 3 clinical trial (NCT02615067) [194]. In 2016, another promising PSMA-based SPECT agent [^99m^Tc]Tc-PSMA-Investigation & Surgery was applied for first-in-human radio-guided surgery (RSG) [195]. The clinical applications of ^99m^Tc radiopharmaceuticals are summarized in Table 12.

#### 5.1.3. Production and Availability

^99m^Tc is a radioactive decay product of ^99^Mo (t_1/2_ = 66h), which is traditionally made in a large nuclear reactor via fission of high-enriched uranium targets (^235^U) [189]. The production of ^99m^Tc in the form of pertechnetate [^99m^Tc]TcO_4_^−^ from the parent ^99^Mo was achieved using the commercially available and transportable ^99^Mo/^99m^Tc generators in nuclear medicine for the preparation of almost all of the ^99m^Tc-based radiopharmaceuticals. Until 2011, the global requirement for ^99^Mo was fulfilled by seven nuclear research reactors. The mandatory shutdowns of these reactors for maintenance or due to breakdowns stopped the global supply in 2009, 2012, and 2013 [189]. To overcome such an unavoidable global crunch in the supply of ^99^Mo, several research efforts were initiated, including the use of linear accelerators and cyclotrons, which utilize electron beam and proton irradiation of solid ^100^Mo targets, respectively [205,206].

### 5.2. Indium-111

#### 5.2.1. General Information

Indium-111 (^111^In; t_1/2_ = 2.8 d) is a SPECT isotope that decays by EC (100%) and low- energy γ-emission (171 keV, 245 keV) [207]. The decay characteristics are summarized in Table 13. Over the decades, ^111^In has been used as the reference standard for SPECT-immuno imaging of Abs [207]. ^110m^In is a PET radioisotope of In with a short half-life (69 min), and it is suitable for tracking short peptides (e.g., octreotide) having faster kinetics [208].

#### 5.2.2. Clinical Practice

In mid-1978, McAffee and Thakur introduced a radiotracer, [^111^In]In-oxine, which could be used to radiolabel leukocytes (white blood cells (WBC)) for the scintigraphic detection of focal infections [209]. In 1985, the FDA approved [^111^In]In-oxine-tagged WBC scans for clinical imaging of inflammatory disease [210]. The reported sensitivity and specificity of these [^111^In]In-WBC scans ranged from 60–100% to 69–92%, respectively, in detecting osteomyelitis, vascular grafts infection, bone infections, etc. [211]. Other than cell labeling, ^111^In was also used in radiolabeling of various peptides, proteins, Abs, and drugs. For example, [^111^In]In-capromab pendetide (ProstaScint^®^) was FDA-approved for immuno-SPECT imaging of PCa [212]; however, the poor tumor-to-background signals limited its routine clinical use [212]. Later, another promising PSMA immuno-SPECT tracer, [^111^In]In-J591 (PSMA-Ab), was developed, and the first clinical trial was reported in 2005 [213]. Clinical trials of [^111^In]In-J591 are underway and associated with the dosimetric projections of RIT with [^90^Y]Y-J591 [214]. Furthermore, in 2018, Heckman et al. demonstrated the first-in-man study using the novel SPECT tracer [^111^In]In-DOTA-girentuximab for intraoperative guidance of renal cell carcinoma resection in patients [215]. Table 14 summarizes the clinical application of ^111^In-labeled radiopharmaceuticals.

#### 5.2.3. Production and Availability

The most common production route of ^111^In in a high yield (222 ± 5 MBq/µA.h) is proton irradiation (21 MeV) of enriched ^112^Cd target via a ^112^Cd (p,2n) ^111^In nuclear reaction using a cyclotron [216].

**Table 14:** Clinical applications of ^111^In-labeled radiopharmaceuticals.

Radiopharmaceuticals	Targets	NCT Number ^	Disease
[^111^In]In-CP04	CCK2R/gastrin	NCT03246659 (Phase I; completed)	Thyroid carcinoma [217]
[^111^In]In-Ch806	gp140, IL-13RA2	NCT00291447 (Phase I; completed)	Neoplasm [218]
[^111^In]In-capromab pendetide (ProstaScint^®^)	PSMA	NCT00992745 (Phase I; completed)	Prostate cancer [219]
[^111^In]In-PSMA (I&T)	PSMA	NCT04300673 (Phase I ongoing)	Prostate cancer [220]
[^111^In]In-DOTA-Girentuximab	Carbonic anhydrase-IX	NCT02497599 (Phase I; status unknown)	Renal cell carcinoma [221]
[^111^In]In-labeled leukocytes	Leukocytes	NCT00026897 (Phase II; completed)	Neoplasm [222]

NCT: National clinical trial, CCK2R/gastrin: Cholecystokinin receptor, gp140: glycoprotein 140, PSMA: Prostate-specific membrane antigen, ^ clinicaltrials.gov and data accessed on August 15, 2023.

## 6. Beta Minus Emitter

### 6.1. Yttrium-90

#### 6.1.1. General Information

Yttrium-90 (^90^Y) is a pure high-energy β^−^ emitter (E_β_^−^_max_ = 2284 keV, E_β_^−^_avg_ = 933 keV), which decays to stable Zr-90 with no accompanying gamma emissions [223] (Table 15). Y-90 has a longer tissue penetration depth of up to 11.8 mm [223]. To date, Y-90 has been radiolabeled with tumor-targeting Abs [224], SSTR-targeting peptides [225], and resins/glass microspheres to treat a variety of tumors [226].

#### 6.1.2. Clinical Application of ^90^Y

The FDA has approved two types of ^90^Y microspheres, TheraSphere^TM^ (glass microspheres) and SIR-spheres^®^ (resin microspheres), to treat unresectable hepatocellular carcinoma and colorectal metastasis, respectively [227,228].

These ^90^Y-microspheres have been used in therapies based on the concept of “radioembolization” (also known as selective internal radiation therapy); it is a promising catheter-based liver-directed therapy approved by the FDA for patients with primary/metastatic liver tumors. It was found that the antitumor effect of ^90^Y-microspheres (glass microspheres, also known as Thersphere^TM^) are related to beta radiations rather than embolization and therefore proven safer/successful for advanced-stage liver cancer [227]. The recent phase III trials of radioembolization of ^90^Y-resin microspheres in patients with HCC demonstrated significantly higher tumor response with respect to standard first-line treatment with Sorafenib. However, these results did not meet the primary endpoint, such as overall survival or the patient’s quality of life. Several Asian guidelines recommend ^90^Y-resin microspheres for HCC treatment based on certain considerations, such as patient selection, treatment planning using accurate dosimetry pre/post-radioembolization, and technical aspects [229,230,231,232].

In 2002, the FDA approved the first anti-CD20 radioimmunoconjugate [^90^Y]Y-Ibritumomab tiuxetan (Zevalin^TM^) for the treatment of advanced B-cell lymphoma as a first line of treatment for rituximab-relapsed or refractory low-grade lymphomas; the overall response rate has ranged from 74% to 82% [233]. Despite the demonstrated immunotherapy efficacy of Zevalin^TM^, it failed commercially due to the underutilized practice by hematologist–oncologists for logistic and economic reasons [234]. In addition, other competitive RIT drugs, such as rituximab (anti-CD20) and second-generation mAbs, undoubtedly contributed to the limited sale of Zevalin^TM^ [235,236].

The development of second-generation mAbs, particularly bispecific Abs (e.g., biotin, IgG-single chain variable fragment), have been utilized in an alternative approach called multi-step pre-targeted RIT to enhance the therapeutic efficacy and to diminish its toxicities [237]. Based on PRIT technology, [^90^Y]Y-DOTA-biotin was developed, which makes a strong conjugate with Abs (streptavidin, avidin) present on the tumor. In 1999, Paganelli et al. published the first clinical preliminary results of [^90^Y]Y-DOTA-biotin for the treatment of high-grade gliomas (*n* = 48) based on biotin–streptavidin chemistry and showed tumor reduction (>25–100%) in 25% of patients; in 16% of these, the response lasted for at least a year [238]. In 2000, a phase II clinical trial of [^90^Y]Y-DOTA-biotin was reported in patients with metastatic colon cancer [239]. Despite evaluating the feasibility, safety, and efficacy of [^90^Y]Y-DOTA-biotin, the immunogenicity of these types of pre-targeting agents have not been addressed, which in turn caused the clinical trials to end in 2005 [240].

Paganelli et al. developed an innovative therapeutic approach called “Intra-operative avidination for radionuclide therapy” (IART^®^) that relies on a biotin–avidin binding system [241]. A phase II study of IART^®^ in 2010 using [^90^Y]Y-DOTA-biotin on breast cancer patients demonstrated its potential use immediately after breast resection, thereby shortening the time course of external beam radiotherapy [241]. In the past decade, several peptide-based ^90^Y-tracers were developed for PRRT, and they are currently under clinical trial. Table 16 summarizes the clinical application of ^90^Y radiopharmaceuticals.

#### 6.1.3. Production and Availability

^90^Y can be produced from the ^90^Sr/^90^Y generator, where the parent isotope is ^90^Sr (t_1/2_ = 29 y), and it can be generated as a by-product in large quantities in U-based nuclear reactions [242]. Commercial availability and the steady supply of Y-90 are advantageous in conducting Y-90-based clinical trials.

**Table 16:** Clinical applications of ^90^Y-labeled radiopharmaceuticals.

Radiopharmaceuticals	Target	NCT Number ^	Disease
[^90^Y]Y-cG250	-	NCT00199875 (Phase I; completed)	Renal and kidney cancer [243]
[^90^Y]Y-hM5A	CEA	NCT00645060 (Phase I; completed) NCT01205022 (Phase I; completed)	Unspecified adult solid tumor [244] Colon and rectal cancer [245]
[^90^Y]Y-hPAM4	MUC1	NCT00603863 (Phase I/II; completed)	Pancreatic [246]
[^90^Y]Y-DOTATOC	SSTR	NCT05568017 (Phase II; ongoing)	Pancreatic neuroendocrine tumor [247]
[^90^Y]Y-edotreotide	SSTR	NCT00006368 (Phase I; completed)	Brain, breast, and lung cancer, lymphoma, melanoma, neoplastic syndrome [248]
[^90^Y]Y-resin microspheres (SIR-spheres^®^)	-	NCT01482442 (Phase III; completed)	Liver carcinoma [249]
[^90^Y]Y- Ibritumomab Tiuxetan (Zevalin^TM^)	CD20 + B cells	NCT01446562 (Phase II; completed)	Follicular lymphoma [250]

NCT: National clinical trial, CEA: Carcinoembryonic antigen, MUC1: Mucin 1, SSTR: Somatostatin receptors, CD20: Cluster of differentiation 20, ^ clinicaltrials.gov and data accessed on 15 August 2023.

### 6.2. Radioisotopes of Rhenium

#### 6.2.1. General Information

Among several radioisotopes of rhenium (Re), ^186^Re and ^188^Re are recognized for their therapeutic potential, and they were used to develop various therapeutic radiopharmaceuticals. In addition to beta emission,^186^Re and ^188^Re also emit low-abundant γ-rays of 137 keV and 155 keV, respectively (Table 17), that permit scintigraphic monitoring and dosimetry calculations via SPECT imaging [251].

Given the two distinct tissue penetration ranges of ^188^Re (11 mm) and ^186^Re (4.5 mm), they can be selectively applied for treating large-sized tumors and small- or mid-sized tumors, respectively [251]. Moreover, to better understand the biodistribution, ^99m^Tc represents a diagnostic match for ^186/188^Re radioisotopes, as both Re and Tc exhibit similar chemical properties [251]. However, ^99m^Tc- and ^188^Re-labeled radiotracers do not always show the same in vivo biodistribution [251].

#### 6.2.2. Clinical Applications of Rhenium Radioisotopes

^188^Re-labeled therapeutic radiopharmaceuticals have been investigated in multiple clinical trials involving primary tumors, bone metastasis, rheumatoid arthritis, and intracoronary β-brachytherapy [252]. In 1998, Maxon et al. evaluated phosphonate-based radiotracer [^188^Re]Re-HEDP for bone pain palliation [253]. Bone pain is a major issue in ~50% of women with breast cancer and 80% of men with PCa. A phase III trial comparing [^188^Re]Re-HEDP with a well-known bone-targeting agent [^223^Ra]RaCl_2_ is ongoing (NCT03458559). The primary objective of this study is to compare the overall survival in patients with PCa metastatic to bone after treatment with [^188^Re]Re-HEDP and [^223^Ra]RaCl_2_. Several Ab fragments have been radiolabeled with ^186/188^Re for RIT. These include alemtuzumab (anti-CD66) in leukemia [254], rituximab (anti-CD20) in lymphoma [255], MN-14 (ant-CEA) in gastrointestinal cancer [256], and bivatuzumab in head and neck cancers [257]. Among them, the evaluation of [^186^Re]Re-bivatuzumab in a variety of diseases (NCT02204033) as a phase I clinical trial has been completed. However, the results are not yet published. Recently, ^188^Re-colloids-based brachytherapy kit (Rhenium-SCT^®^) became commercially available to treat basal cell carcinoma or squamous cell carcinoma, particularly to the face and neck, where surgery and radiotherapy are either not possible or refused by patients (NCT05135052) [258]. Several preliminary clinical reports have demonstrated that this innovative epidermal therapy is effective in 98% of melanoma patients even after a single application [259]. The clinical investigations of Re radiopharmaceuticals are summarized in Table 18.

#### 6.2.3. Production and Availability

^188^Re is routinely produced in high specific activity by a ^188^W/^188^Re generator, like the ^99m^Tc generator [260]. On other hand, ^186^Re is most commonly produced in apparent specific activity of 111–148 GBq/mg at the Missouri Research Nuclear Reactor [261].

**Table 18:** Clinical applications of ^188^Re-labeled radiopharmaceuticals.

Radiopharmaceuticals	Targets	NCT Number ^	Disease
[^188^Re]Re-HEDP vs. [^223^Ra]RaCl_2_	Bone metastasis	NCT03458559 (Phase III; ongoing)	Prostate cancer metastatic to bone [262]
^[186^Re]Re-labeled bivatuzumab	VEGF-A	NCT02204046 (Phase I; completed), NCT02204059 (Phase I; completed), NCT02204033 (Phase I; completed)	Adenocarcinoma [263] Non-small cell lung carcinoma [264] Head and neck neoplasm [265]
Rhenium-SCT^®^	Skin lesions	NCT05135052 (Phase not applicable; ongoing)	Non-melanoma skin cancer [258]
[^186^Re]Re-nanoliposome	-	NCT01906385 (Phase I/II; ongoing)	Glioma [266]

NCT: National clinical trial, VEGF-A: Vascular Endothelial Growth Factor Receptors-A, ^ clinicaltrials.gov and data accessed on 15 August 2023.

### 6.3. Holomium-166

^166^Ho is not only a β^−^ emitter but also a gamma emitter; it is one of the lanthanide radionuclides that can be imaged using SPECT and MRI [267,268]. ^166^Ho is a theranostic radionuclide with favorable physical decay characteristics, including a sufficient half-life of 26.6 h, an average emission energy of (E_βav_) of 670 keV, a soft tissue penetration range of 8.7 mm, and a low-energy γ-emission (80.5 keV, 6%) for SPECT imaging [267,268]. Being a lanthanide with its paramagnetic properties, ^166^Ho-labeled drugs enable the visualization and quantification of the biodistribution of drugs in the tumor tissues by means of SPECT and MRI [268]. In 1991, Murphy et al. first investigated the potential possibility of ^166^Ho microspheres for the internal radiation therapy of hepatic tumors in rabbits [269]. In 2010, Smith et al. investigated the first ^166^Ho-based liver radioembolization, which was followed by growing interest in this treatment possibility, as evidenced by the increasing number of publications in the last few years [268]. In terms of clinical applications, ^166^Ho-microspheres serve as an alternative to existing ^90^Y microspheres to treat liver tumors, with potential advantages of the shorter half-life of ^166^Ho (t_1/2_ = 26.6 h) compared to ^90^Y (t_1/2_ = 64 h) along with its quantification by MRI [268]. Additional information on ^166^Ho- radiopharmaceuticals have been discussed in a recent review by Klaassen et al. [270]; therefore, we have kept the discussion extremely short.

### 6.4. Lutetium-177

#### 6.4.1. General Information

^177^Lu is currently the most important and highly valuable theranostic β^−^/γ-emitting radionuclide in nuclear medicine across the globe [271]. ^177^Lu has a long half-life (t_1/2_ = 6.7d) and decays to ^177^Hf by emitting medium-energy cytotoxic β^−^ particles, with the most abundant β^−^ particles (78%) having a maximum energy of 0.497 MeV (Table 19) [271]. Furthermore, the co-emission of γ-photons (112.9 keV, 208.5 keV) enables the visualization and quantification (dosimetry) of the biodistribution of ^177^Lu- radiopharmaceuticals using SPECT [271].

#### 6.4.2. Clinical Applications

Since 2000, ^177^Lu-labeled somatostatin analogues have been utilized in PRRT for the treatment of inoperable or metastatic NETs [272]. ^177^Lu-labeled somatostatin has six- to seven-fold higher affinity for SSTR2 compared with its ^90^Y-loaded counterpart [272]. Several preclinical and clinical studies have been conducted on the therapeutic effectiveness of ^177^Lu-based radiopharmaceuticals in last two decades [273]. In 2005, the first-in-human proof-of-concept study was published on endoradiotherapy with [^177^Lu]Lu-PSMA-I & T, which was found to be promising in patients with castration-resistant and metastatic prostate cancers [274].

In 2017, the results of a clinical phase 3 trial (NETTER-1) involving 229 patients randomized to either PRRT using [^177^Lu]Lu-DOTATATE (7.4 GBq every 8 weeks) or a long-acting release (LAR) formulation of octreotide (control groups) to treat patients with midgut NET were released [275,276]. The groups receiving [^177^Lu]Lu-DOTATATE had a significantly higher response rate (18%) and longer progression-free survival (65.2%) at 20 months compared to the controls, with 10.8 and 3%, respectively. [^177^Lu]Lu-DOTATATE treatment yielded a clinically significant improvement in progression-free survival as a primary end point as well as an improvement in the median survival of 11.7 months [276]. Overall, the treatment was well tolerated with grade 3 or 4 adverse events, which were similar in both the groups. No evidence of renal toxicities was observed among patients in the [^177^Lu]Lu-DOTATATE groups [275,276]. In 2018, the U.S. Food and Drug Administration (FDA) and the European Medicines Agency (EMA) approved [^177^Lu]Lu-DOTATATE (Lutathera ^®^; Novartis company) for mid gut NET. In the same year, [^177^Lu]Lu-PSMA-617 was proposed for the treatment of metastatic castration-resistant prostate cancer (mCRPC). The results of a phase 2 trial (TheraP) demonstrated a significant decline (>50%) in PSA in the groups treated with [^177^Lu]Lu-PSMA-617 compared to the standard treatment using Carbazitaxel, which eventually led to FDA and EMA approvals under the name of Pluvicto^®^(Novartis). The clinical use of ^177^Lu-radiopharmaceuticals has been increased in the past few years, including [^177^Lu]Lu-FAPI-46 [277,278] and combination therapy with ^90^Y-labeled peptides and chemotherapy. Several clinical trials of ^177^Lu-based radio theranostics are underway, as listed in Table 20.

#### 6.4.3. Production and Availability

There are two common independent ways to produce large-scale ^177^Lu in nuclear reactors. The first is a direct production route (also known as carrier added or c.a.) based on neutron irradiation of ^176^Lu via ^176^Lu (n, γ) ^177^Lu nuclear reaction in medium–high-energy reactors [271,288]. The second approach is an indirect production route (also known as non-carrier added or n.c.a) based on neutron irradiation of ^176^Yb target via ^176^Yb (n, γ) ^177^Yb →^177^Lu in high-energy flux reactors [288]. The advantage of the direct production route is that it can create large quantities of ^177^Lu (740–1110 GBq) using ^176^Lu; however, the major concern is the co-emission of small amounts of long-lived radioactive impurity of ^177m^Lu along with “useful” ^177^Lu. Additionally, only a part of the target matrix (or carrier) of ^176^Lu is converted into the desired ^177^Lu, which cannot be chemically isolated as they are the isotopes of the same element; this therefore decreases its specific activity [288].

On the other hand, the indirect approach using highly enriched ^176^Yb (>98%) produces high specific activity (>2.96 TBq/mg) non-carrier-added ^177^Lu; however, this process requires a suitable method for the radiochemical separation of ^177^Lu from ^176^Yb, which is quite challenging, especially in large-scale or industrial settings, to meet the surging demand [271,288]. ^177^Lu can also be produced in cyclotron using deuteron beams (<6 MeV); however, this is less explored due to the low production yield [288].

## 7. Alpha-Particle-Emitting Radiopharmaceuticals

Alpha radiations are better suited for the treatment of small metastasis due to their short tissue penetration range and high LET per micrometer of tissue compared to β^−^-emitting radionuclide via double strand DNA breaks in cancerous cells, while sparing nearby healthy tissues [21]. However, due to the early stages of development of alpha-targeted radionuclide therapy, most clinical trials continue to use beta-emitting radionuclide therapy rather than alpha-emitting radionuclide therapy.

### 7.1. Radioisotopes of Bismuth

#### 7.1.1. General Information

Two promising medically relevant isotopes of bismuth (Bi) with mixed α/β^−^ emission properties are ^212^Bi and ^213^Bi [289]. ^212^Bi undergoes β^−^ decay (64%) to ^212^Po (α-emitter) and α-decay (36%) to ^208^Tl (β^−^ emitter). Both daughters of ^212^Bi (^212^Po and ^208^Tl) further decay to the stable ^208^Pb [289]. Moreover, the emission of high-energy γ-rays through the decay of ^208^Tl (2.6 MeV) necessitates appropriate shielding to avoid radiation exposure, making it a less favorable choice over ^213^Bi [289].

^213^Bi is considered a magic bullet in targeted radionuclide therapy. The isotope predominantly undergoes β^−^ decay (97.8%) to the pure α-emitter ^213^Po, whereas the remaining ^213^Bi (2.2%) undergoes α- decay to beta-emitter ^209^Tl [289]. Both of these daughter nuclei (^213^Po, ^209^Tl) decay to ^209^Pb, which further decays to long-lived ^209^Bi (essentially stable) [289]. In addition, ^213^Bi also emits γ-radiation (440 keV) that can be employed for SPECT imaging [289]. Overall, each ^213^Bi decay delivers only one α particle (5.9–8.4 MeV) [289]. The nuclear decay properties of Bi radionuclides are given in Table 21.

#### 7.1.2. Clinical Applications of ^213^Bismuth

In 2002, Joseph et al. reported the first proof-of-concept phase I study demonstrating the anti-leukemic effect of ^213^Bi conjugated with anti-leukemia Ab HuM195 (Lintuzumab) to treat leukemia patients [290]. In a subsequent clinical study (phase I/II) in 2010, complete remission was seen in acute myeloid leukemia patients with sequential administration of [^213^Bi]Bi-HuM195 (37 MBq/Kg) and the chemotherapy drug cytarabine (Table 22) [291]. The promising clinical results with [^212^Bi]Bi-mAb-TAT initiated its use to treat other cancers, including melanoma, NETs, and glioma [292,293].

In the last two decades, research efforts have facilitated the development of ^213^Bi-based peptide conjugates for PRRT study. In 2014, Kratochwil et al. reported the first and only radiopeptide therapy with [^213^Bi]Bi-DOTATOC on NET patients, which was refractory to β^−^ therapy with ^90^Y/^177^Lu-DOTATOC [294]. The results indicated that TAT could induce considerable and long-lasting remission in both the primary tumor and liver metastases [294]. Another tracer, [^213^Bi]Bi-PSMA-617, was reported in metastatic castration-resistant PCa patients [295]. The remarkable drop in prostate-specific antigen levels from 237 µg/L to 43 µg/L after [^213^Bi]Bi-PSMA-617 treatment showed the great potential of TAT using [^213^Bi]Bi-PSMA-617 over conventional β^−^ radionuclide therapy. In addition, TAT may be able to break the radioresistant effect of β^−^ emitters [295]. In the past few years, different clinical trials have used ^213^Bi-carrying radiopharmaceuticals for the treatment of various diseases. Although the outcomes were encouraging, further investigations are needed to ensure its safety and efficacy in the clinic.

**Table 21:** Decay characteristics of radioisotopes of Bismuth ^#^.

Isotope	Half-Life (t_1/2_)	Decay Characteristics	Parent Nuclides and Their Daughter Nuclides	Energies (MeV)		E_γ;_ keV (Intensity%)
				E_α_ (MeV)	E_β_^−^ (MeV)	
^212^Bi	61 min	β^−^ = 64% α = 36%	^212^Bi ^212^Po ^208^Tl ^208^Pb (stable)	^212^Bi-6.1 ^212^Po-8.8	^212^Po-0.769 ^208^Tl-0.557	^212^Bi-727.3 (6.6) ^208^Tl-277.4 (6.3), 510.8 (22.6), 583.2 (84.5), 763 (1.8), 860.6 (12.4), 2614.5 (99.2)
^213^Bi	45.6 min	β^−^ = 98% α = 2%	^213^Bi ^213^Po ^209^Tl ^209^Pb ^209^Bi (essentially stable)	5.9 (^213^Bi) 8.4 (^213^Po)	^213^Bi-1400 ^209^Tl-2000 ^209^Pb-600	^213^Bi-440 (25.9)

^#^ Data on ^212^Bi are from [296] and data on ^213^Bi are from [297]. Please refer to Scheme 2.

#### 7.1.3. Production and Availability

Many α-emitter radionuclides are produced from naturally occurring heavy α radionuclides, including U, radium (Ra), and actinium (Ac). The clinical amount of ^213^Bi is obtained from its parent radionuclide ^225^Ac (t_1/2_ = 9.9 d) as a ^225^Ac/^213^Bi generator [298]. The parent isotope ^225^Ac is obtained from the decay of ^229^Th (t_1/2_ = 7317 y), which in turn originates from a decay chain of fissile materials of ^233^U [298]. The relatively long half-life of the parent radionuclide ^225^Ac allows shipment of the ^225^Ac/^213^Bi generator to any radiopharmaceutical facility located even long distances away and permits in-house generation of ^213^Bi for radiolabeling purposes over weeks to months. However, the limited global production of ^229^Th and the concern for the non-proliferation of the fissile product of ^233^U restricted the commercial supply of ^225^Ac stocks to produce ^213^Bi-labeled radiopharmaceuticals [293]. An alternate route to producing ^225^Ac is using proton irradiation of ^226^Ra targets via ^226^Ra (p,2n) ^225^Ac in a cyclotron; still, the presence of hazardous ^222^Rn poses serious limitations in clinical translation and waste disposal [293].

**Table 22:** Clinical applications of ^213^Bi-labeled radiopharmaceuticals.

Radiopharmaceuticals	Targets	NCT Number ^	Disease
[^213^Bi]Bi-M195	CD33	NCT00014495 (Phase I/II completed)	Leukemia, myelodysplastic syndromes [299]

NCT: National clinical trial, ^ clinicaltrials.gov and data accessed on 15 August 2023.

### 7.2. Actinium-225

#### 7.2.1. General Information

^225^Ac is one of the promising therapeutic isotopes for α-RIT of cancer. It decays to six principal intermediate radionuclide progenies (^221^Fr, ^217^At, ^213^Bi, ^213^Po, ^209^Tl, ^209^Pb) before reaching the stable ^209^Bi [300]. Overall, ^225^Ac decay (t_1/2_ = 9.9 d) contributes to the emission of four α-, three β^−^, and two principal γ-emissions (218 keV; ^221^Fr, 440 keV; ^213^Bi), from which recognizable ^225^Ac results as a “nanogenerator.” ^225^Ac is also considered an in vivo generator of ^213^Bi and an alternative to ^213^Bi-based TAT, presumably because of the four α- emissions and its longer half-life compared to ^213^Bi (t_1/2_ = 45.6 min) [300]. The decay characteristics of Bi radionuclides are given in Table 23.

^225^Ac-based radiopharmaceuticals are prone to the redistribution of daughter progenies, particularly ^213^Bi, which can induce renal toxicity and dose-limiting toxicity to other organs [300]. Moreover, dosimetry is essential using an isotope with a similar half-life and chelation chemistry to ^225^Ac (e.g., Ln^3+^) to track the biodistribution of ^225^Ac accurately. A handful of clinical trials of ^225^Ac are underway.

#### 7.2.2. Clinical Applications of Actinium-225

In 2011, the first clinical study of α therapy was reported showing the anti-leukemic effect of [^225^Ac]-lintuzumab in acute myeloid leukemia patients (>60 y) [301]. Motivated by the initial findings, several clinical trials (phase I/II), including a dose-escalation study of [^225^Ac]Ac-lintuzumab combined with low-dose chemotherapeutic drugs (e.g., mitoxantrone, cladribine), have been initiated (NCT03867682 [258]).

By 2018, a multicenter phase I study using [^225^Ac]Ac-FPI-1434 (NCT03746431) was designed to treat solid tumors from non-cell lung, prostate, and breast carcinomas [302]. Recently, a clinical study reported by Kratochwil et al. demonstrated the remarkable anti-tumor effect of [^225^Ac]Ac-PSMA-617 (100 kBq/kg) in 81% of metastatic castration-resistant PCa patients [303]. Additional clinical trials are warranted to further investigate the anti-tumor potential of [^225^Ac]Ac-PSMA-617 TAT in men with prostate cancer (NCT04597411). The clinical investigation of ^225^Ac radiopharmaceuticals is summarized in Table 24.

#### 7.2.3. Production and Availability

Currently, the clinical supply of ^225^Ac is produced from ^229^Th generators (t_1/2_ = 7340 y), which are obtained from the parent ^233^U (t_1/2_ = 160,000y) [300]. ^229^Th generators are available at the Oak Ridge National Laboratory USA, the Institute of Transuranium Elements, Germany, and the Institute of Physics and Power, Russia [300]. However, as of 2008, the approximate total worldwide production of ^225^Ac accounts for only 68 GBq/year, which can support only several hundred patients per year. Therefore, large-scale production of ^225^Ac is needed. Alternative production routes are being explored, including proton irradiation of ^226^Ra targets, which could produce sufficient quantities of ^225^Ac due to the relatively high reaction cross-section; however, the handling of ^226^Ra (t_1/2_ = 1600 y) is challenging [300].

To date, the accelerator-based production route involves high-energy proton irradiation (>100 MeV) of natural thorium (^232^Th), and it could serve as another potential path for the future production of ^225^Ac. This method may yield twenty times greater quantities of ^225^Ac than the current annual production worldwide [310].

### 7.3. Radioisotopes of Lead

#### 7.3.1. General Information

Lead (^212^Pb; t_1/2_ = 10.6 h) is a β^−^-emitting radionuclide that decays to ^212^Bi (t_1/2_ = 61 min), which decays by mixed α/β- particle emission [311]. Importantly, ^212^Pb also emits imageable γ-radiation (238.6 keV) that has the potential to image ^212^Pb-labeled radiopharmaceuticals directly via SPECT imaging (Table 25). Moreover, ^203^Pb is a γ-emitting analogue of ^212^Pb, and it is considered an ideal SPECT imaging isotope for the estimation of an accurate dosimetry for ^212^Pb-labeled therapeutic radiopharmaceuticals [311].

#### 7.3.2. Clinical Practice

During 2014–2018, Meredith et al. performed several clinical studies using therapeutic [^212^Pb]Pb-TCMC-trastuzumab in HER2 expressing malignancy, with promising outcomes, including improved safety, tolerability, and therapeutic efficacy [313,314,315]. Delpassand et al. investigated [^212^Pb]Pb-DOTAMTATE (Alpha Medix^TM^) for the treatment of inoperable SSTR-NETs, which could be superior to the gold standard β^−^-emitting [^177^Lu]Lu-DOTATATE radiopharmaceutical [316]. The clinical investigation of Pb radiopharmaceuticals has been detailed in Table 26.

#### 7.3.3. Production and Availability

^212^Pb is commonly produced from the decay chain of a ^228^Th (t_1/2_ = 1.9 y) generator, followed by its elution in 2M HCl using a cation exchange column with a maximum yield of 85% [319]. At high radioactivity (>37 MBq), however, the radiolytic damage of the cation exchange resin in the ^228^Th generator increases the back pressure and decreases the yield [319]. To circumvent this, an alternative generator using ^224^Ra (t_1/2_ = 3.7 d) was designed, which serves as a source of either ^212^Bi or its parent nuclide ^212^Pb [319]. The ^224^Ra/^212^Pb generator could elute ^212^Pb with a radioactivity up to ~600 MBq (16 mCi) [319]. Currently, ^212^Pb is mainly supplied by OranoMed and Oak Ridge National Laboratory [319]. McNeil et al. established a production protocol of ^203^Pb via proton irradiation of either natural thallium (Tl) or enriched ^203^Tl in a TR13 (13 MeV) cyclotron to create a ^228Th^/^212^Pb generator for ^212^Pb [312].

### 7.4. Radioisotopes of Radium

#### 7.4.1. General Information

Ra has several radioisotopes, of which ^223^Ra and ^224^Ra are of considerable interest to the medical field as bone-seeking α-emitters for TAT [320]. ^223^Ra (t_1/2_ = 11.4 d) is an α-emitter that decays to ^207^Pb via six intermediate progenies (^219^Rn, ^215^Po,^211^Pb, ^211^Bi, ^211^Po, ^207^Tl) and delivers four α particles and two beta particles (Table 27) [320]. However, ^223^Ra faces challenges in quantitative imaging because of the limited abundance of short-range gamma photons (<2%) [321]. Several research studies are ongoing to investigate its dosimetry approach [322,323,324].

^224^Ra is a pure α-emitter that decays via a series of six daughter nuclides (^220^Rn, ^216^Po,^212^Pb, ^212^Bi, ^212^Po, ^208^Tl) and emit overall four alpha particles and two beta particles before stabilizing to ^208^Pb [325]. ^224^Ra emits abundant gamma emissions at 241 keV that can be employed for SPECT imaging [325]. ^224^Ra (3.6d) has a shorter half-life than ^223^Ra (11.4d), but its decay profile and biokinetics are like ^223^Ra [307].

**Table 27:** Decay characteristics of radioisotopes of Radium ^#^.

Isotope	Half-Life (t_1/2_)	Decay Characteristics	Parent and Daughter Nuclides	Energy (MeV)		E_γ_; keV (Intensity%)
				E_α max_	E_β-max_	
^223^Ra	11.4 d	α = 100%	^223^Ra ^219^Rn ^215^Po ^211^Pb ^211^Bi ^211^Po ^207^Tl ^207^Pb (stable)	^223^Ra-5.78 ^219^Rn-6.88 ^215^Po-7.53 ^211^Bi-6.68 ^211^Po-7.59	^211^Pb-0.45 ^211^Bi-0.01 ^207^Tl-0.49	144.27 (3.36) 154.2 (5.84) 323.8 (4.06) 328.2 (2.85)
^224^Ra	3.6d	α = 100%	^224^Ra ^220^Rn ^216^Po ^212^Pb ^212^Bi ^212^Po ^208^Tl ^208^Pb (stable)	^224^Ra-5.7 ^220^Rn-6.3 ^216^Po-6.8 ^212^Bi-6.1 ^212^Po-8.8	^212^Pb-0.1 ^212^Bi-0.8 ^208^Tl-0.6	241(4.1%)

^#^ Data on ^223^Ra are from [326,327], and data on ^224^Ra are from [328]. Please refer to Scheme 2.

#### 7.4.2. Clinical Practice

From mid-1940 to 1990, [^224^Ra]RaCl_2_ of high doses (up to 140 MBq) was used to treat different bone and joint diseases, mainly in Germany, but this practice was abandoned for technical and commercial reasons [329]. During 2000–2005, the use of [^224^Ra]RaCl_2_ (low dose up to 10 MBq) was revived to treat ankylosing spondylitis patients, but this was discontinued in 2005 due to the enhanced risk of malignant disease following injection [330,331]. One of the potential drawbacks of ^224^Ra is the release of progeny β^−^-emitting ^212^Pb with a significant half-life of 10.6 h, which could cause unwanted non-target exposure [330]. Therefore, alternative delivery strategies are of considerable interest, which could promote the retention of the daughter nuclides or mitigate their recoiling spread.

During 2007–2015, several preclinical studies investigated brachytherapy using ^224^Ra-loaded diffusing α-emitter radiation therapy (DaRT) wires or seeds, which minimizes the damage to surrounding normal tissues [332]. The first-in-human clinical study based on DaRT was reported in 2020 and involved the implantation of ^224^Ra seeds to treat squamous cancers of the skin and head [333]. Complete response to the ^224^Ra-DaRT treatment was observed in 22 of the 28 patients; the remaining 6 patients showed only a partial response (>30% tumor reduction) [333]. Like ^224^Ra, ^223^Ra was also studied for the treatment of bone skeletal metastasis. The first clinical study (phase I) in prostate and breast cancer patients was reported by Nilsson et al. in 2005 [334]. Later, the favorable clinical results (phase II/III) of [^223^Ra]RaCl_2_ to treat metastatic PCa led to FDA approval of [^223^Ra]RaCl_2_ (Xofigo^®^; Bayer) in 2013 [335,336]. Several clinical trials of ^223^Ra-based radionuclide therapy in combination with chemotherapy (docetaxel, paclitaxel), hormonal therapy (abiraterone, enzalutamide), and immunotherapy are ongoing (Table 28).

#### 7.4.3. Production and Availability

^223^Ra is mainly produced from ^227^Ac/^227^Th generators, where ^223^Ra is separated from ^227^Ac/^227^Th mother radionuclides using separation columns [300,350]. On the other hand, ^224^Ra is usually produced from a ^228^Th generator, where ^228^Th is immobilized on actinide resin, which allows regular elution of ^224^Ra in 1M HCl [351].

### 7.5. Thorium-227

#### 7.5.1. General Information

^227^Th (a progenitor of ^223^Ra) is an α-emitting radionuclide that decays to ^223^Ra, which further decays by a series of α and β^−^ emissions before stabilizing to ^207^Pb [352] (Table 29). ^227^Th can be readily chelated with 3, 2-hydroxypyridone-N-oxide (HOPO). When ^227^Th is conjugated with tumor-targeting moieties, they are collectively called targeted thorium-227 conjugates (TTCs) [353].

#### 7.5.2. Clinical Practice

There are four clinical trials listed for ^227^Th-based TTCs registered in the US National Library of Medicine. These trials are based on ^227^Th-labeled anti-PSMA-HOPO (Bay 2315497) and ^227^Th-labeled anti-mesothelin-HOPO for the treatment of PCa (NCT03724747) [355] and mesothelioma (Bay 2287411), respectively. The remaining two trials are based on ^227^Th-labeled epratuzumab-HOPO (Bay 1862864) and ^227^Th-labeled trastuzumab-HOPO (Bay 2701439) to treat CD22-positive non-Hodgkin’s lymphoma and HER2-positive breast or gastric cancers, respectively (Table 30). ^89^Zr-labeled HOPO has the potential to serve as a PET surrogate for TTCs, which could support the clinical development of novel TTCs by providing crucial pharmacokinetic and pharmacodynamic information [356].

#### 7.5.3. Production and Availability

^227^Th is produced as a decay product of the parent β^−^ emitter ^227^Ac (t_1/2_ = 21.8 year) [357]. The longer half-life of ^227^Th (t_1/2_ = 18.7 days) allows for the shipment of cGMP-grade ^227^Th solution worldwide [357].

**Table 30:** Clinical applications of ^227^Th-labeled radiopharmaceuticals.

Radiopharmaceuticals	Targets	NCT Number ^	Disease
[^227^Th]Th-anti PSMA (BAY2315497)	PSMA	NCT03724747 (Phase I; ongoing)	Metastatic castration-resistant prostate cancer [355]
[^227^Th]Th-anti Mesothelin (BAY2287411)	Mesothelin	NCT03507452 (Phase I; completed)	Advanced recurrent serous ovarian, malignant peritoneal mesothelioma, pancreatic adenocarcinoma [358]
[^227^Th]Th-trastuzumab (BAY2701439)	HER2+	NCT04147819 (Phase I; ongoing)	Cancer with HER2 + expression [359]
[^227^Th]Th-epratuzumab (BAY1862864)	CD22	NCT02581878 (Phase I; completed)	Non-Hodgkin lymphoma [360]

NCT: National clinical trial, PSMA: Prostate-specific membrane antigen, HER2+: Human epidermal growth factor 2, CD22: Cluster of differentiation 22, ^ clinicaltrials.gov and data accessed on 15 August 2023.

### 7.6. Radioisotopes of Astatine

#### 7.6.1. General Information

Astatine-221 (^211^At) is an α-emitting therapeutic radionuclide that decays into two branches either by α-emission (42%) to ^207^Bi (t_1/2_ = 33.9 y) or by EC (58%) to ^211^Po (t_1/2_ = 516 ms); both eventually decay to a stable ^207^Pb [361]. Each decay yields one α particle and the emission of characteristic X-rays (70–90 keV) through the decay of ^211^Po and could be used for SPECT imaging and quantification of ^211^At [361]. ^209^At (t_1/2_ = 5.4 h) is another isotope that predominantly decays by β^+^ emission (96%) and has been introduced as a theranostic pair to ^211^At (Table 31) [361]. ^211^At is a more attractive radionuclide than other α-emitting radionuclides because of its suitable half-life of 7.2 h, the absence of long-lived and/or toxic progenies, and its feasibility to be produced in decent quantities [361].

#### 7.6.2. Clinical Practice

Although ^211^At-labeled TAT agents were discovered more than 30 years ago, only a few clinical studies using ^211^At-labeled Abs have been published. Zalutsky et al. reported on the application of ^211^At-labeled chimeric anti-tenascin mAb 81C6 (71–347 MBq) in recurrent brain tumor patients with an encouraging median survival time of 52 weeks compared to 23 weeks reported for recurrent glioblastoma multiforme patients treated with best care [362]. Another clinical study of intraperitoneal α particle therapy was reported using [^211^At]At-MX35(Fab) in relapsed ovarian cancer patients [363]. The results showed that there was no apparent radiation-induced toxicity discovered in patients for up to 12 years and no decreased tolerance to relapse therapy. The clinical investigation of ^211^At-labeled radiopharmaceuticals is summarized in Table 32.

#### 7.6.3. Production and Availability

The most common route is the cyclotron/accelerator-based production of ^211^At through alpha irradiation of ^209^Bi (natural Bi) via a ^209^Bi(α,2n)^211^At nuclear reaction [364,365]. However, only a limited number of cyclotrons with α-beam and with > 25 MeV energy are available in the field, limiting the overall ^211^At availability [364]. Other methods include the use of ^211^Rn/^211^At generators [366]. One of the potential advantages of using ^211^Rn/^211^At generators is the longer half-life of ^211^Rn (t_1/2_ = 14.6 h) compared with ^211^At (t_1/2_ = 7.2 h), facilitating wider distribution of ^211^At.

**Table 32:** Clinical applications of Astatine-211-labeled radiopharmaceuticals.

Radiopharmaceuticals	Targets	NCT Number ^	Disease
Sodium Astatide ([^211^At]NaAt)	-	NCT05275946 (Phase I; ongoing)	Thyroid cancer [367]
[^211^At]At- 81C6	Glial fibrillary acidic protein	NCT00003461 (Phase I/II; completed)	Metastatic cancer, brain and central nervous system tumors, neuroblastoma [368]
[^211^At]At- bc8-b10	CD45	NCT04083183 (Phase I/II; ongoing) NCT03670966 (Phase I/II; ongoing)	Non-malignant neoplasm [369] Acute lymphoblastic leukemia in remission [370]
[^211^At]At-OKT-B10	CD3	NCT04466475 (Phase I; ongoing)	Plasma cell myeloma [371]

NCT: National clinical trial, CD45: Cluster of differentiation, CD3: Cluster of differentiation 3, ^ clinicaltrials.gov and data accessed on 15 August 2023.

## 8. Conclusions

In summary, the development of radiometal-based radiopharmaceuticals, including their production, purification, bifunctional chelating agents, and biomarker discoveries, have significantly advanced the application of various radiometals in medicine in the last two decades. Both radiometal-based imaging and radionuclide therapy are changing the lives of patients on a daily basis due to the advancements made in the last 20 years. The field of α-emitting radiotherapy is emerging. Several clinical trials are currently under investigation. Further advances in the production and availability of these α-emitters along with the management of radioactive progeny should permit the cost-effective clinical adoption of TAT compared to traditional chemotherapeutics. Indeed, the future of the radiometal-based radiopharmaceutical industry appears to be very bright.

## Figures and Tables

**Scheme 1:**
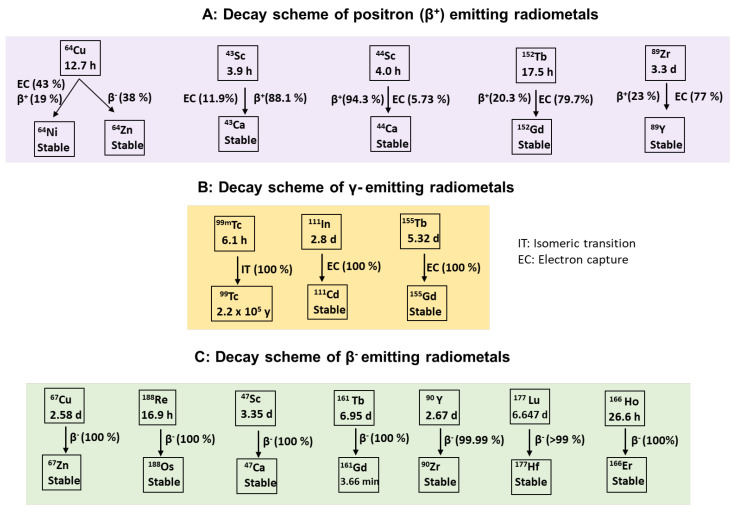
Decay scheme for various radionuclides.

**Scheme 2:**
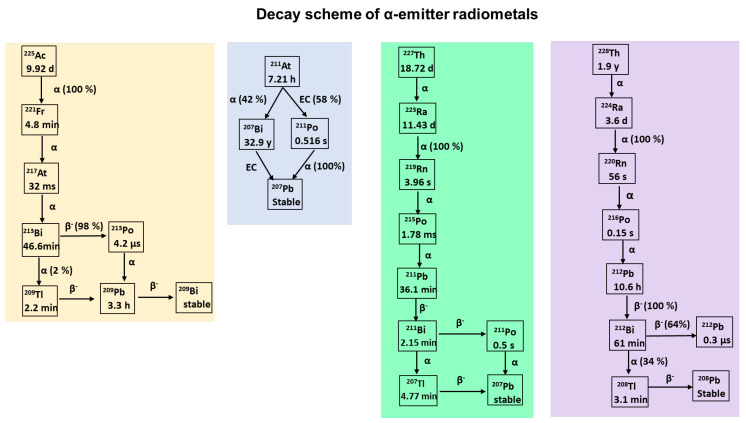
Decay scheme for various α-emitting radionuclides.

**Table 1:** Decay characteristics of copper radioisotopes used in radiopharmaceuticals ^#^.

Isotope	Half-Life (t_1/2_)	Decay Characteristics	Energies		E_γ_; keV (Intensity %)
			E_β_^+^_avg_ (keV)	E_β_^−^_avg_ (keV)	
^60^Cu	23.7 min	β^+^ = 93% EC = 7%	970	-	1332.5 (88) 1791.6 (45.4) 826.4 (21.7)
^61^Cu	3.33 h	β^+^ = 61% EC = 39%	500	-	282.95 (12.7) 656 (10.4)
^62^Cu	9.76 min	β^+^ = 97% EC = 2%	2910	-	511 (194)
^64^Cu	12.70 h	β^−^ = 38.5% β^+^ = 17.6% EC = 43.9%	278	191	1345.77 (0.475)
^67^Cu	61.83 h	β^−^ = 100%	-	141	184.57 (48.7)

^#^ Data on ^60/61/64/67^Cu are from [24] and data on ^62^Cu are from [25]. Please refer to Scheme 1A.

**Table 2:** Clinical applications of various ^64/67^Cu-labeled radiopharmaceuticals.

Radiopharmaceuticals	Targets	NCT Number ^	Disease [Ref.]
[^64^Cu]Cu-ATSM	Hypoxia-targeted	NCT03951337 (Phase II; ongoing)	Rectum cancer [32]
[^64^Cu]Cu-DOTA-trastuzumab	HER2^+^	NCT02827877 (Phase II; ongoing)	Breast cancer [56]
[^64^Cu]Cu-DOTA-M5A	CEA	NCT05245786 (Early phase I; ongoing)	Rectal cancer [57]
[^64^Cu]Cu- SarTATE	SSTR	NCT04438304 (Phase II; ongoing)	Neuroendocrine tumors [58]
[^64^Cu]Cu-TP3805	VPAC1	NCT02603965; (Phase I; completed)	Prostate cancer [59]
[^64^Cu]Cu-DOTA-AE105	uPAR	NCT02139371 (Early phase I; completed)	Breast, prostate, and bladder cancer [60]
[^64^Cu]Cu-SAR-bisPSMA	PSMA	NCT04839367 (Phase I; completed) NCT05249127 (Phase I/II; ongoing)	Prostate neoplasms [61] Recurrent prostate neoplasm [62]
[^67^Cu]Cu- SarTATE	SSTR	NCT03936426 (Phase I/IIa; completed), NCT04023331 (Phase I/IIa; ongoing)	Meningioma [63] Neuroblastoma [64]
[^64/67^Cu]Cu-SAR-bisPSMA	PSMA	NCT04868604 (Phase I/IIa; ongoing)	Castration-resistant prostate cancer [65]

NCT: National clinical trial, uPAR: Urokinase plasminogen activator receptor, CEA: Carcinoembryonic antigen, VPAC1: Vasoactive intestinal polypeptide, SSTR: Somatostatin receptors, PSMA: Prostate-specific membrane antigen, ^ clinicaltrials.gov and data accessed on 15 August 2023.

**Table 3:** Decay characteristics of the three main radioisotopes of gallium ^#^.

Isotope	Half-Life (t_1/2_)	Decay Characteristics	Energy	E_γ;_ keV (Intensity %)
			E_β+avg_ (keV)	
^66^Ga	9.49 h	β^+^ = 57%	1750	1039.22 (37)
		EC = 43%		
^67^Ga	3.26 d	EC = 100%	-	93.31 (38.81)
^68^Ga	67.71 min	β^+^ = 88.91%	829.5	1077.34 (3.22)
		EC = 11.09%		

^#^ Data on ^66/67/68^Ga are from [78]. Please refer to Scheme 1A.

**Table 7:** Decay characteristics of commonly used radioisotopes of scandium ^#^.

Isotope	Half-Life (t_1/2_)	Decay Characteristics	Energies		E_γ_; keV (Intensity%)
			E_β_^+^_avg_ (keV)	E_β_^−^_avg_ (keV)	
^43^Sc	3.9 h	β^+^ = 88%	476	-	372(23)
		EC = 12%			
^44^Sc	4.0 h	β^+^ = 94%	632	-	1157(100)
		EC = 6%			
^47^Sc	3.35 d	β^−^ = 100%	-	162	159(68)

^#^Data on ^43/44/47^Sc are from [150]. Please refer to decay Scheme 1A.

**Table 8:** Decay characteristics of leading radioisotopes of terbium ^#^.

Isotope	Half-Life (t_1/2_)	Decay Characteristics	Energy			E_γ_; keV (Intensity %)
			E_β+avg_ (keV)	E_α avg_ (keV)	E_β-avg_ (keV)	
^149^Tb (α-therapy)	4.12 h	α = 16.7%	730	3967	-	165 (26), 352 (29)
		β^+^ = 7.1%				388.6 (18)
		EC = 76.2%				652.1 (16)
^152^Tb (PET)	17.5 h	β^+^ = 17%	1080	-	-	344.3 (65)
		EC = 83%				586.3(9.4)
^155^Tb (SPECT)	5.32 d	EC = 100%	-	-	-	86.55 (32)
						105.3 (25)
^161^Tb (β^−^/MAE therapy)	6.89 d	β^−^ = 100%	-	-	154	25.65 (23)
						48.92 (17)
						74.57 (10)

^#^ Data on ^149/152/155/161^Tb are from [167]. Please refer to decay Scheme 1A.

**Table 9:** Clinical applications of terbium-labeled radiopharmaceuticals under investigation.

Radiopharmaceuticals	Targets	NCT Number ^	Disease
[^161^Tb]Tb-DOTA-LM3	SSTR2	NCT05359146 (Early phase 1; recruiting)	Neuroendocrine neoplasia or gastroenteropancreatic neuroendocrine tumor [179]
[^161^Tb]Tb-PSMA-I&T	PSMA	NCT05521412 (Phase I/II; recruiting)	Prostate cancer or metastatic castration-resistant prostate cancer [180]

NCT: National clinical trial, SSTR2: Somatostatin-targeting receptor 2, PSMA: Prostate-specific membrane antigen, ^ clinicaltrials.gov and data accessed on 15 August 2023.

**Table 10:** Decay characteristics of PET isotopes of zinc ^#^.

Isotope	Half-Life (t_1/2_)	Decay Characteristics	E_β+avg_ (keV)	E_γ_; keV (Intensity%)
^62^Zn	9.26 h	β^+^ = 8.2%	259	508 (15), 550 (15)
				600 (26)
^63^Zn	38.47 min	β^+^ = 93%	992	670 (8)
				960 (7)
^65^Zn	243.9 d	β^+^ = 98%	142.5	1110 (50.6)

^#^ Data on ^62/63/65^Zn are from [184]. Please refer to Scheme 1A.

**Table 11:** Decay characteristics of Technetium-99 m ^#^.

Isotope	Half-Life (t_1/2_)	Decay Characteristics	E_γ_; keV (Intensity %)
^99m^Tc	6.0 h	IT = 100%	140.51 (98.6)

^#^ Data on ^99m^Tc are from [188]. Please refer to Scheme 1B.

**Table 12:** Clinical applications of ^99m^Tc-labeled radiopharmaceuticals.

Radiopharmaceuticals	Targets	NCT Number ^	Disease
[^99m^Tc]Tc-tilmanocept	Lymph node	NCT02201420 (Phase II; completed)	Kaposi’s sarcoma [196]
[^99m^ Tc]Tc-EC20	Folate	NCT01689714 (Phase II; completed)	Ovarian or recurrent endometrial carcinoma [197]
[^99m^ Tc]Tc-Tetrofosmin	-	NCT02971319 (Phase II; completed)	Glioma [198]
[^99m^ Tc]Tc-Sestamibi	-	NCT05042687 (Phase not applicable)	Breast cancer [199]
[^99m^ Tc]Tc-HYNIC TOC EDAA	SSTR	NCT02691078 (Phase II completed)	Neuroendocrine tumors [200]
[^99m^ Tc]Tc-MP-1404	PSMA	NCT02615067 (Phase III completed)	Prostate cancer [201]
[^99m^ Tc]Tc-MP-1404 [^99m^ Tc]Tc-MP-1405	PSMA	NCT01261754 (Phase I; completed)	Prostate cancer [202]
[^99m^ Tc]Tc-PSMA I&S	PSMA	NCT04832958 (Phase II; ongoing)	Prostate cancer [203]
[^99m^ Tc]Tc-labeled albumin in macroaggregates (MAA) and in microspheres (B20)	-	NCT01186263 (Phase II; completed)	Colorectal cancer, liver metastasis [204]

NCT: National clinical trial, PSMA: Prostate-specific molecular antigen, SSTR: Somatostatin-targeting receptor, ^ clinicaltrials.gov and data accessed on 15 August 2023.

**Table 13:** Decay characteristics of radioisotopes of indium ^#^.

Isotope	Half-Life (t_1/2_)	Decay Characteristics	E_β_^+^_avg_ (keV)	E_γ_; keV (Intensity %)
^110m^In	69.1min	β^+^ = 61.3%	1011	657.75(97.74)
		EC = 38%		
^111^In	2.8 d	EC = 100%	-	245.35(94.1)

^#^ Data on ^110m/111^In are from [78]. Please refer to Scheme 1B.

**Table 15:** Decay characteristics of Yttrium-90 ^#^.

Isotope	Half-Life (t_1/2_)	Decay Characteristics	Energy	
			E_β_^−^_max_ (MeV)	E_β_^−^_avg_ (MeV)
^90^Y	64.0 h	β^−^ = 100%	2.284	0.933

^#^ Data on ^90^Y are from [223]. Please refer to Scheme 1C.

**Table 17:** Decay characteristics of ^186/188^Re ^#^.

Isotope	Half-Life (t_1/2_)	Decay Characteristics	E_β_^−^_avg_ KeV	E_γ_; keV (Intensity%)
^186^Re	90 h	β^−^ = 92.59%	346.7	137.15 (9.47)
		EC = 7.41%		106 (12.1)
^188^Re	17.0 h	β^−^ = 100%	763	155.04 (15.49)
				478 (1.076%)

^#^ Data on ^186/188^Re are from [251]. Please refer to Scheme 1C.

**Table 19:** Decay characteristics of Lutetium-177 ^#^.

Isotope	Half-Life (t_1/2_)	E_β-max_ (keV)	E_ϒ_; keV (Intensity%)
^177^Lu	6.647 d	497 (78.6%) 384 (9.1%) 176 (12.2%)	208 (11%) 113 (6.6%)

^#^ Data on ^177^Lu are from [271]. Please refer to decay Scheme 1C.

**Table 20:** Clinical applications of ^177^Lu-labeled radiopharmaceuticals.

Radiopharmaceuticals	Targets	NCT Number ^	Disease [Ref.]
[^177^Lu]Lu-PSMA-617 (PLUVICTO ^®^)	PSMA	NCT03511664 (Phase III; ongoing)	Metastatic prostate cancer [279]
^223^Ra + [^177^Lu]Lu-PSMA- I & T	PSMA	NCT05383079 (phase II; recruiting)	Metastatic castration-resistant prostate cancer [280]
Cabozantinib in Combination With [^177^Lu]Lu- DOTATATE (LuTATE)	SSTR2	NCT05249114 (Phase Ib; ongoing)	Neuroendocrine tumors [281]
capecitabine (CAP)/temozolomide (TEM) + [^177^Lu]Lu- DOTATATE (LuTATE)	SSTR	NCT02358356 (phase II; completed)	Mid gut or pancreatic neuroendocrine tumors [282]
[^177^Lu]Lu- DOTATATE (Lutathera)	SSTR2	NCT03206060 (phase II; ongoing)	Pheochromocytoma/Paraganglioma, neuroendocrine tumor [283]
[^177^Lu]Lu-Edotreotide	SSTR	NCT04919226 (phase III; ongoing)	Gastroenteropancreatic neuroendocrine tumors [284]
[^177^Lu]Lu- catalase	-	NCT05985278 (Early phase 1; ongoing)	Advanced malignant neoplasm [285]
[^177^Lu]Lu-EB-FAPI	FAP	NCT05400967 (Early phase 1; ongoing)	Metastatic tumors [286]
[^177^Lu]Lu-DOTA-girentuximab	Carbonic anhydrase IX	NCT02002312 (phase II; completed)	Metastatic clear cell renal cancer [287]

NCT: National clinical trial, SSTR: Somatostatin receptors, FAP: Fibroblast activation protein, ^ clinicaltrials.gov and data accessed on 15 August 2023.

**Table 23:** Decay characteristics of Actinimum-225 ^#^.

Isotope	Half-Life (t_1/2_)	Decay Characteristics	^225^Ac and Daughter Nuclides	Energies (MeV)		E_γ_; keV (Intensity%)
				E_αmax_	E_β_^−^ _max_	
^225^Ac	9.9 d	α = 100%	^225^Ac ^221^Fr ^217^At ^213^Bi ^213^Po ^209^Tl ^209^Pb ^209^Bi (stable)	^225^Ac-5.8 ^221^Fr-6.3 ^217^At-7.1 ^213^Bi-5.9 ^213^Po-8.4	^213^Bi-0.492 ^209^Tl-0.178 ^209^Pb-0.198	^213^Bi-100 (1) ^221^Fr-218 (11.4) ^213^Bi-440 (26) ^209^Tl-1567 (99.7)

^#^ Data on ^225^Ac are from [300]. Please refer to Scheme 2.

**Table 24:** Clinical application of Actinium-225-labeled radiopharmaceuticals.

Radiopharmaceuticals	Targets	NCT Number ^	Disease
[^225^Ac]Ac-lintuzumab with Venetoclax	BCL-2	NCT03867682 (Phase I/II; ongoing)	Acute and relapsed myeloid leukemia [304]
[^225^Ac]Ac-DOTA-Daratumumab	CD38	NCT05363111 (Phase I; ongoing)	Recurrent plasma cell myeloma [305]
[^225^Ac]Ac-FPI-1434	IGF-1R	NCT03746431 (Phase I/II; ongoing)	Advanced solid tumor, endometrial cancer, ovarian, cervical cancer [306]
[^225^Ac]Ac-DOTA-M5A	CEA	NCT05204147 (Phase I; ongoing)	Advanced and metastatic cancer [307]
[^225^Ac]Ac-PSMA-617	PSMA	NCT04597411 (Phase I; ongoing)	Castration-resistant prostate cancer [308]
[^225^Ac]Ac-J591	PSMA	NCT03276572 (Phase I; ongoing)	Prostate cancer [309]

NCT: National clinical trial, BCL-2: B-cell lymphoma 2, CD38: Cluster of differentiation 38, IGF-1R: Type 1 insulin-like growth factor receptor, CEA: Carcinoembryonic antigen, PSMA: Prostate-specific membrane antigen, ^ clinicaltrials.gov and data accessed on 15 August 2023.

**Table 25:** Decay characteristics of lead ^#^.

Isotope	Half-Life (t_1/2_)	Decay Characteristics	Parent and Daughter Nuclides	Energies		E_γ_; keV; (Intensity%)
				E_αmax_ (MeV)	E_β- max_ (MeV)	
^203^Pb	51.9h	EC = 100%	^203^Tl (stable)	-	-	279 (81)
^212^Pb	10.6h	β^−^ = 100%	^212^Pb ^212^Bi ^212^Po ^208^Tl ^208^Pb (stable)	^212^Bi-6.1 ^212^Po-8.8	^212^Pb-0.102 ^212^Bi-0.769 ^208^Tl-0.557	^212^Pb-238.6 (43.6) ^212^Bi-727.3 (6.6) ^208^Tl-277.4 (6.3), 510.8 (22.6), 583.2 (84.5), 763 (1.8), 860.6 (12.4), 2614.5 (99.2)

^#^ Data on ^203^Pb are from [312] and data on ^212^Pb are from [296]. Please refer to Scheme 2.

**Table 26:** Clinical applications of ^212^lead-212-labeled radiopharmaceuticals.

Radiopharmaceuticals	Targets	NCT Number ^	Disease
[^212^Pb]Pb-DOTAMTATE (Alpha Medix^TM^)	SSTR	NCT05153772 (Phase II; ongoing)	Neuroendocrine tumors [317]
[^212^Pb]Pb-TCMC-Trastuzumab	HER2+	NCT01384253 (Phase I; completed)	Breast, ovarian, peritoneal, pancreatic, and stomach neoplasm [318]

NCT: National clinical trial, SSTR: Somatostatin receptors, HER2+: Human epidermal growth factor 2, ^ clinicaltrials.gov and data accessed on 15 August 2023.

**Table 28:** Clinical applications of ^223^Ra in combination with other therapies.

Radiopharmaceuticals	Targets	NCT Number ^	Disease
[^223^Ra]Ra-dichloride	Skeletal metastasis	NCT01833520 (Phase II; completed)	Sarcoma [337]
[^223^Ra]Ra-dichloride + Niraparib	PARP inhibitor	NCT03076203 (Phase I; completed)	Prostate cancer metastatic to bone, stage IV prostate cancer, hormone refractory prostate cancer [338]
[^223^Ra]Ra-dichloride + Abiraterone, Prednisone/Prednisolone	CYP17 inhibitor	NCT02043678 (Phase III; active)	Prostate cancer [339]
[^223^Ra]Ra-dichloride + Enzalutamide	AR inhibitor	NCT02199197 (Phase II; completed) NCT03305224 (Phase II; ongoing)	Prostate cancer, [340], bone metastatic prostate cancer [341]
[^223^Ra]Ra-dichloride + Denosumab	Cytokine RANKL	NCT02366130 (Phase II; completed)	Breast carcinoma [342]
[^223^Ra]Ra-dichloride + Paclitaxel	Tubulin	NCT02442063 (Phase I; completed)	Neoplasm, bone disease [343]
[^223^Ra]Ra-dichloride + Docetaxel	P300	NCT03574571 (Phase III; ongoing)	Prostate cancer [344]
[^223^Ra]Ra-dichloride + Leuprolide acetate,	GnRH-receptor agonist	NCT03361735 (Phase II; ongoing)	Prostate cancer [345]
[^223^Ra]Ra-dichloride + Pembrolizumab	PDL-1	NCT03093428 (Phase II; ongoing)	Prostate cancer [346]
[^223^Ra]Ra-dichloride + Atezolizumab	PDL-1	NCT02814669 (Phase I; completed)	Castration-resistant prostate cancer [347]
Alpha-DaRT seeds (^224^Ra containing 316LVM tubes)	Implantation sites	NCT04002479 (Phase not applicable) NCT03970967 (Phase not applicable)	Metastatic pancreatic cancer [348] Metastatic breast cancer [349]

NCT: National clinical trial, PARP: Polyadenosine diphosphate-ribose polymerase, CYP17: Cytochrome P450 17 α-hydroxylase/17,20-lyase, AR inhibitor: Androgen receptors, RANKL: Receptor activator of nuclear factor-kB ligand, GnRH-receptor agonist: Gonadotropin-releasing hormone, PDL-1: Programmed cell death ligand-1, ^ clinicaltrials.gov and data accessed on 15 August 2023.

**Table 29:** Decay characteristics of thorium-227, which follow a decay chain of radium-223 ^#^.

Isotope	Half-Life (t_1/2_)	^227^Th and Daughter Nuclides		E_αmax_ (MeV)	
				E_αmax_	E_β_^−^_max_
^227^Th	18.7 d	α = 100%	^227^Th ^223^Ra ^219^Rn ^215^Po ^211^Pb ^211^Bi ^207^Tl ^211^Po ^207^Pb (stable)	^227^Th-5.9 ^223^Ra-5.7 ^219^Rn-6.8 ^215^Po-7.4 ^211^Bi-6.6 ^211^Po-7.6	^211^Pb-0.4 ^211^Bi-0.6 ^207^Tl-0.5

^#^ Data on ^227^Th are from [354]. Please refer to Scheme 2.

**Table 31:** Decay characteristics of Astatine-221 ^#^.

Isotope	Half-Life (t_1/2_)	Decay Characteristics	^211^At and Daughter Nuclides	E_αmax_ (MeV)	E_γ_; keV
^211^At	7.2 h	α = 42% EC = 58%	^207^Bi ^211^Po ^207^Pb (stable)	5.87 7.45	^211^At-687 ^211^Po-569.7, 897.8

^#^ Data on ^211^At are from [361]. Please refer to Scheme 2.

## Data Availability

Data are contained within the article.

## Abbreviations

Ab	=	Antibody
Ac	=	Actinium
AML	=	Acute myeloid leukemia
At	=	Astatine
Bi	=	Bismuth
CD8	=	Cluster of differentiation 8
CD38	=	Cluster of differentiation 38
CD20	=	Cluster of differentiation 20
CEA	=	Carcinoembryonic antigen
CERN	=	European Council for Nuclear Research
Cu	=	Copper
DaRT	=	Diffusing alpha-emitters radiation therapy
EC	=	Electron capture
EGFR	=	Epidermal growth factor receptor
FAPI	=	Fibroblast activation protein inhibitor
FDA	=	Food and Drug Administration
Ga	=	Gallium
GBM	=	Glioblastoma Multiforme
Ge	=	Germanium
GMP	=	Good manufacturing practice
HER2	=	Human epidermal growth factor 2
HOPO	=	2-hydroxypyridone-N-oxide
IART^®^	=	Intra-operative avidination for radionuclide therapy
ISOLDE	=	Isotope separation on-line
LET	=	Linear energy transfer
mAb	=	Monoclonal antibody
MAE	=	Meitner–Auger electrons
mCRPC	=	Metastatic castrate-resistant prostate cancer
MUC1	=	Mucin-1
NCT	=	National clinical trial
NET	=	Neuroendocrine tumor
Pb	=	Lead
PCa	=	Prostate cancer
PDL-1	=	Programmed cell death ligand-1
PET	=	Positron emission tomography
PRRT	=	Peptide receptor radionuclide therapy
PSA	=	Prostate-specific antigen
PSMA	=	Prostate-specific membrane antigen
Ra	=	Radium
Re	=	Rhenium
RIT	=	Radioimmunotherapy
Sc	=	Scandium
SPECT	=	Single photon emission computed tomography
SSTR2	=	Somatostatin-targeting receptor 2
SUV	=	Standardized uptake value
TAT	=	Targeted alpha therapy
Tb	=	Terbium
Tc	=	Technetium
Th	=	Thorium
Tl	=	Thallium
TRT	=	Targeted radionuclide therapy
TTC	=	Targeted thorium conjugates
U	=	Uranium
VEGF	=	Vascular endothelial growth factor
Y	=	Yttrium
Zn	=	Zinc
Zr	=	Zirconium
